# Thy1-ApoE4/C/EBPβ double transgenic mice act as a sporadic model with Alzheimer’s disease

**DOI:** 10.1038/s41380-024-02565-x

**Published:** 2024-04-24

**Authors:** Zhengjiang Qian, ZhiHao Wang, Bowei Li, Xin Meng, Zhonghua Kuang, Yanjiao Li, Yongfeng Yang, Keqiang Ye

**Affiliations:** 1 Faculty of Life and Health Sciences, Brain Cognition and Brain Disease Institute (BCBDI), Shenzhen Institute of Advanced Technology (SIAT), Chinese Academy of Sciences, Shenzhen, 518055 Guangdong China; 2 Department of Neurology, Renmin Hospital of Wuhan University, Wuhan, Hubei Province 430060 China; 3 Shenzhen Institute of Advanced Technology, University of Chinese Academy of Science, Shenzhen, Guangdong Province 518055 China; 4 Paul C. Lauterbur Research Center for Biomedical Imaging, Institute of Biomedical and Health Engineering, Shenzhen Institute of Advanced Technology (SIAT), Chinese Academy of Sciences, Shenzhen, 518055 Guangdong China

**Keywords:** Neuroscience, Molecular biology

## Abstract

Early onset familial Alzheimer’s disease (FAD) with APP, PS1/2 (presenilins) mutation accounts for only a small portion of AD cases, and most are late-onset sporadic. However, majority of AD mouse models are developed to mimic the genetic cause of human AD by overexpressing mutated forms of human APP, PS1/2, and/or Tau protein, though there is no Tau mutation in AD, and no single mouse model recapitulates all aspects of AD pathology. Here, we report Thy1-ApoE4/C/EBPβ double transgenic mouse model that demonstrates key AD pathologies in an age-dependent manner in absence of any human APP or PS1/2 mutation. Using the clinical diagnosis criteria, we show that this mouse model exhibits tempo-spatial features in AD patient brains, including progressive cognitive decline associated with brain atrophy, which is accompanied with extensive neuronal degeneration. Remarkably, the mice display gradual Aβ aggregation and neurofibrillary tangles formation in the brain validated by Aβ PET and Tau PET. Moreover, the mice reveal widespread neuroinflammation as shown in AD brains. Hence, Thy1-ApoE4/C/EBPβ mouse model acts as a sporadic AD mouse model, reconstituting the major AD pathologies.

## Introduction

Alzheimer’s disease (AD), a progressive neurodegenerative disorder, is the most common cause of dementia. The featured hallmarks of AD pathology include diffuse and neuritic plaques, which are mainly composed of the amyloid-β (Aβ) peptide, and neurofibrillary tangles (NFT), consisted of filamentous aggregates of hyperphosphorylated and truncated Tau proteins [1]. These misfolded and aggregated proteins bind to pattern recognition receptors on microglia and astroglia, and trigger an innate immune response and release of inflammatory mediators, which contribute to disease progression [2]. Moreover, loss of neuronal synaptic density and synapses signifies another crucial feature of the disease that takes place before extensive neuronal loss [3, 4]. Notably, the memory and cognitive decline observed in AD patients correlates better with the synaptic pathology than either senile plaques or NFT [5–9].

The risk of AD is 60-80% dependent on heritable factors, of which ApoE4 reveals the strongest association with the disease [10]. ApoE4 significantly increases the risk for both early-onset and late-onset AD [11, 12]. ApoE4 increases AD risk in a dose-dependent manner, individuals that are homozygous for ApoE4 alleles are eight times more likely to develop AD than are homozygotes for ApoE3. However, ApoE4 is neither necessary nor sufficient to cause AD [13]. The proposed mechanisms are multifactorial, including both Aβ-dependent effects, i.e. modulation of Aβ levels, aggregation, neurotoxicity and neuroinflammation, and Aβ-independent effects, i.e. neuronal development, brain activity and lipid metabolism [14]. While Aβ is continuously generated in the brain, it is efficiently eliminated under physiological conditions. Markedly, Aβ clearance is slower in ApoE4-TR mice than ApoE3-TR mice [15]. In addition, young ApoE4 carriers show an increased inflammatory response that may relate to AD risk later in life [16]. ApoE is physiologically expressed in glia cells in the brain [17], but it is induced in neurons under stresses [18–20], and neuronal ApoE4 is neurotoxic [21]. Single-cell sequence shows that human neurons in the brain physiologically express ApoE [22]. Further, neuronal ApoE upregulates MHC-1 expression to drive neurodegeneration in AD [23].

C/EBPβ (CCAAT/enhancer-binding protein β), an important transcription factor in the differentiation and maturation of adipocytes, mediates inflammatory cytokine expression [24] and is progressively escalated in neurons with aging [25]. It mediates AEP (asparagine endopeptidase, also called legumain, gene name: *LGMN*) expression in an age-dependent manner [26]. AEP acts as a δ-secretase that primarily cleaves both APP and Tau at N585 and N368 residues, respectively, stimulating Aβ production and amyloid pathology and NFT. Deletion of AEP or C/EBPβ from AD mouse models substantially diminishes AD pathologies, restoring the cognitive functions [27–29]. Aβ and inflammatory cytokine IL-6 additively activate C/EBPβ [30]. Recently, we report that C/EBPβ functions as a crucial transcription factor for ApoE and regulates its mRNA levels in an age-dependent way [31]. Knockout of C/EBPβ in AD mouse models diminishes ApoE expression and Aβ pathologies, whereas overexpression of C/EBPβ accelerates AD pathologies. Remarkably, C/EBPβ selectively promotes more ApoE4 expression than ApoE3 in human neurons, in alignment with higher activation of C/EBPβ in human AD brains with ApoE4/4 compared to ApoE3/3 [31]. Furthermore, neuronal ApoE4 and 27-hydroxycholesterol activate C/EBPβ/δ-secretase pathway via neuronal secreted Aβ or inflammatory cytokines. 27-hydroxycholesterol strongly activates C/EBPβ/δ-secretase pathway in human ApoE4-TR mice and triggers AD pathologies and cognitive deficits [32]. Consistent with its role in above findings, C/EBPβ elevation in microglia also exacerbates Tau-driven AD pathology [33]. In addition to *ApoE4* and *LGMN*, we showed that C/EBPβ mediates *APP*, *MAPT* and *BACE1* mRNA expression in neurons as well. The escalated APP and Tau proteins are subsequently cleaved by active AEP, resulting in Aβ accumulation and Tau aggregation and impaired synaptic plasticity. ApoE4 is elevated in neurons under stress and augmented in human AD neurons [18–20, 31], and C/EBPβ is highly expressed in aged neurons and AD brains [26]. Therefore, we developed a neuronal specific Thy1-ApoE4/C/EBPβ transgenic mice to simulate the events in human AD brain, and found neuronal ApoE4 drives C/EBPβ, triggering AD pathology cascade in this mouse model [34].

Dozens of AD mouse models have been developed to mimic the genetic cause of human AD, and the transgenic mice overexpress mutated forms of human APP, PS1/2, and/or Tau. Many of the transgenic AD models exhibit Aβ aggregates, neuronal loss, gliosis and Tau pathology, associated with cognitive impairments, but no single AD model recapitulates full spectrum of AD pathology [35]. Hence, proper sporadic AD models are urgently needed to study the mechanisms underlying AD pathogenesis, and environmental risk factors that cause sporadic AD, as well as to test the therapeutic effects of AD drug-candidates on neuropathology and cognitive functions. In this study, we employed the standard clinical diagnostic criteria for AD patients, which include MMSE (Mini-mental Stage Examination, cognitive functions), magnetic resonance imaging (MRI) analysis, cerebro-spinal fluid (CSF) Aβ42/Aβ40 ratio, p-Tau and total Tau levels, and Aβ PET and Tau PET, to fully characterize Thy1-ApoE4/C/EBPβ Tg mice and compare with 3xTg AD mice side-by-side. We found that this mouse model mimics human AD pathologies tempo-spatial distribution and propagation in the absence of any APP or PS1/2 mutation. Therefore, this mouse is a long-awaited innovative sporadic AD mouse model, and neuronal ApoE4 activates C/EBPβ/δ-secretase pathway which might underlie the key dominant molecular mechanism driving AD pathogenesis.

## Results

### Age-dependent learning and memory deficits in 3xTg and Thy1-ApoE4/C/EBPβ Tg mice

**Fig. 1:**
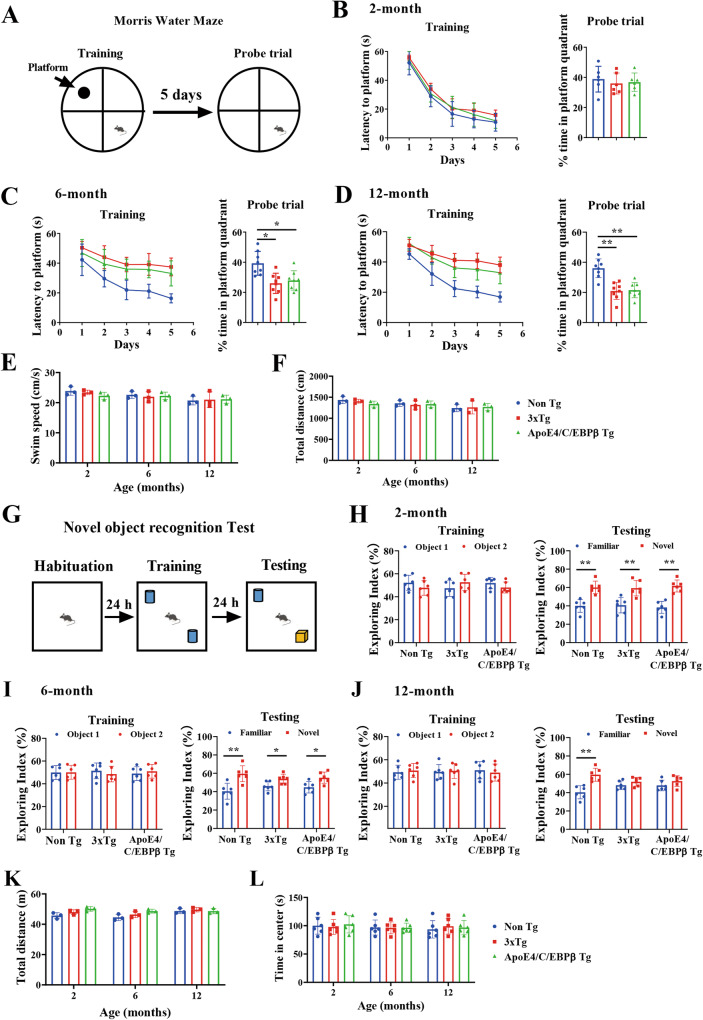
Age-dependent spatial learning and memory deficits in 3xTg and Thy1- ApoE4/C/EBPβ Tg mouse. **A** Diagram showing experiment of Morris Water Maze (MWM). Latency to mount the submerged platform (left panel) and percentage of time in platform quadrant (right panel) in MWM assay with mice at 2-month **B**, 6-month **C** and 12-month **D** of age (*n* = 6, *p < 0.05, compared with Non Tg mice). Swim speed **E** and total distance **F** in probe trial was similar among the mice at all ages. Motor function is unchanged in 3xTg and Thy1- ApoE4/C/EBPβ Tg mouse. **G** Diagram showing experiment of Novel Object Recognition Test (NORT). Exploring index of mice at 2-month **H**, 6-month **I** and 12-month **J** of age. (*n* = 6, *p < 0.05, **p < 0.01, compared with Non Tg mice). Total distance **K** and time spend in the center **L** were comparable to these mice at all ages.

To investigate whether the neuronal ApoE4/C/EBPβ double transgenic mice mimic AD patients and display progressive learning and memory defects and act as a sporadic AD mouse model, we compared it with broadly employed genetic AD mouse model 3xTg, a triple-transgenic model harboring human patient-derived mutants PS1 (M146V), APP (Swe) and tau (P301L) transgenes [36]. Morris Water Maze (MWM) assay showed that both mouse models exhibited cognitive deficits at 6 months and got worse at 12 months of age. However, at 2 months old, neither of them demonstrated any defects. The swim speeds or travel distances were comparable among the groups, which were not changed from 2 months to 12 months of age (Fig. 1A–F), indicating that Thy1-ApoE4/C/EBPβ Tg mice motor functions are normal. Novel Object Recognition test (NORT) revealed that the Exploring Index remained the same among the control mice, 3xTg and Thy1-ApoE4/C/EBPβ Tg mice during the training course from 2 to 12 month of age. During the Testing phase, the Exploring Index showed both 3xTg and Thy1-ApoE4/C/EBPβ Tg failed to show any enhanced interest in exploring the novel object compared to the familiar object at 12 months, though they both demonstrated similar exploring interest to the novel object to the control mice at 2 and 6 months of age. Again, the total travel distance and time in the center were similar among the groups from 2 to 12 months (Fig. 1G–L). Hence, Thy1-ApoE4/C/EBPβ double Tg mice demonstrate the same age-dependent learning and memory defects as 3xTg AD mice.

C/EBPβ is a transcription factor for APP, MAPT and BACE1 [34], which are subsequently cleaved by AEP into APP N585 and Tau N368 and BACE1 N294, respectively, stimulating Aβ and p-Tau pathologies [27, 28, 37]. AEP enzymatic assay with the brain lysates validated AEP was highly activated in 6 and 12-month old 3xTg and Thy1-ApoE4/C/EBPβ AD mouse brains (Supplementary Fig. 1A). To validate whether neuronal ApoE4 activates C/EBPβ/AEP signaling in Thy1-ApoE4/C/EBPβ Tg mice, we conducted immunoblotting (IB) analysis, and found that both C/EBPβ and p-C/EBPβ levels were higher in 3xTg and Thy1-ApoE4/C/EBPβ mice than control mice at both 6 and 12 months old. Consequently, the downstream target AEP activation echoed the upstream transcription factor activities. Accordingly, APP N585 and Tau N368 were prominently cleaved by active AEP. The relative band’s intensities in IB were quantified (Supplementary Fig. 1B, C).

### Age-dependent brain volume reduction in 3xTg and Thy1-ApoE4/C/EBPβ Tg mice

**Fig. 2:**
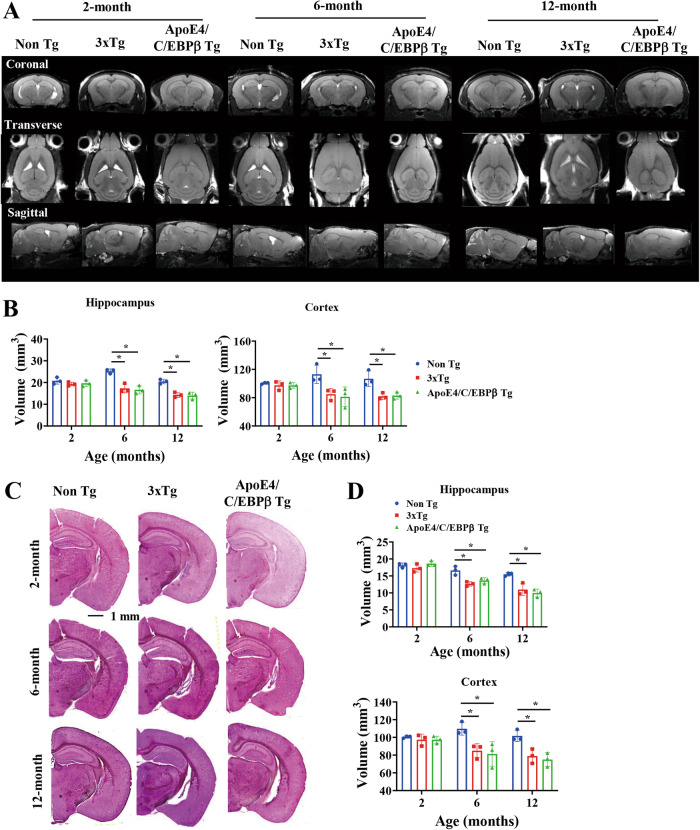
The brain volume is reduced in an age-dependent manner in 3xTg and Thy1- ApoE4/C/EBPβ Tg mouse. **A** Representative MRI images at coronal, transverse and sagittal sections of Non Tg, 3xTg and Thy1-ApoE4/C/EBPβ Tg mice at 2-, 6- and 12-month of age. **B** Relative quantification of hippocampal and cortical volumes by MRI. (*n* = 3, *p < 0.05, compared with Non Tg). **C** Representative Nissl staining of brain sections of 3xTg and Thy1-ApoE4/C/EBPβ Tg mice at 2-, 6- and 12-month of age. Scale bar, 1 mm. **D** Relative quantification of hippocampal and cortical volumes by Nissl staining. (*n* = 3, *p < 0.05, compared with Non Tg).

AD patient brains display cerebral atrophy and white matter changes by antemortem MRI reflect underlying neuropathology [38]. A longitudinal multimodal in vivo molecular imaging study of the 3xTg AD mouse model shows progressive early hippocampal volume loss [39]. Accordingly, we conducted an MRI assay of ApoE4/C/EBPβ double Tg mice and found that both 3xTg and Thy1-ApoE4/C/EBPβ Tg exhibited an age-dependent brain volumes reduction at 6 and 12 months old in both hippocampus and cortex regions. By contrast, the MRI signals remained comparable among the groups at 2 months of age (Fig. 2A, B). Nissl staining and quantification of the brain section from these mice showed that the brain volumes of 3xTg and Thy1-ApoE4/C/EBPβ Tg mice decrease as compared to control mice in both regions from 6 to 12 months of age (Fig. 2C, D), correlating with MRI findings. Hence, these two AD mouse models demonstrate the brain structure atrophy in the same age.

To explore whether neurodegeneration, the cellular mechanism underlying the brain volume reduction is accountable for this effect, we performed immunofluorescent (IF) co-staining with TUNEL (red) and NeuN (green) on the brain sections and found that gradual TUNEL fluorescent signals escalation in both 3xTg and Thy1-ApoE4/C/EBPβ Tg mice compared to control mice starting from 6 months old and apoptotic activities increased up to approximately 30% of neurons at 12 months old in both hippocampus and cortex regions (Supplementary Fig. 2A, B). Next, we also conducted IB assay and found that the levels of PSD95 and synaptophysin, two well-characterized postsynaptic and presynaptic biomarkers, were significantly decreased at 12 months old in both 3xTg and Thy1-ApoE4/C/EBPβ Tg mice versus control mice, indicating extensive synaptic loss in the brains of both AD mouse models (Supplementary Fig. 2C, D). These molecular events temporally fit with the brain structural loss by MRI.

### Age-dependent Aβ PET signal escalation in the brains of 3xTg and Thy1-ApoE4/C/EBPβ Tg mice

**Fig. 3:**
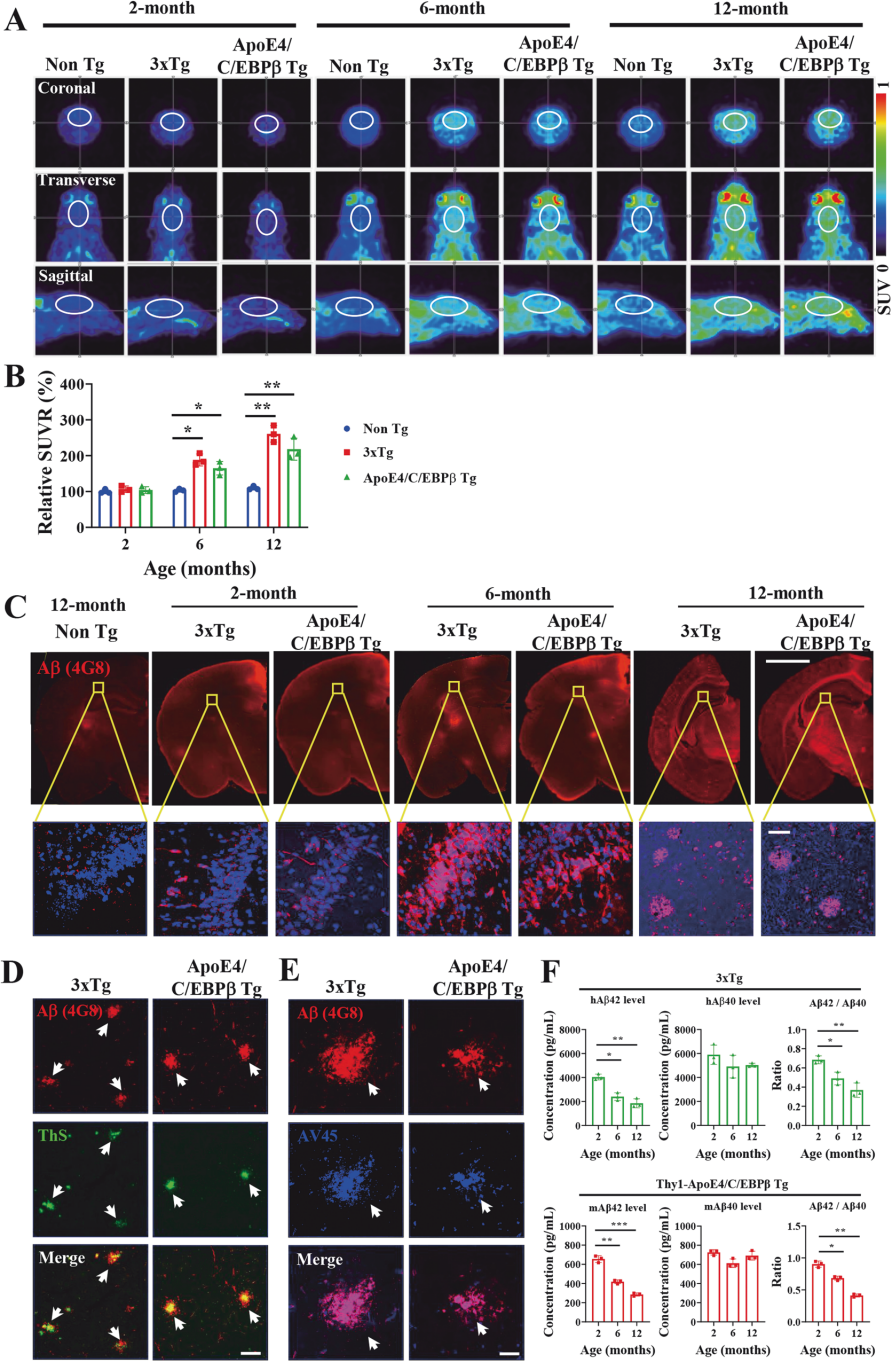
Age-dependent increase of Aβ pathology in 3xTg and Thy1-ApoE4/C/EBPβ Tg mice. **A** Representative Aβ PET images showing the Aβ plaque deposition in the brains of 3xTg and Thy1-ApoE4/C/EBPβ Tg mice at 2-, 6- and 12-month of age. The brain region was labeled with white circle. **B** Relative quantification of Aβ PET standard uptake value ratio (SUVR) in (A) (n = 3 mice, **p < 0.01, compared with Non Tg). **C** Representative of Aβ immunostaining images in the hippocampus of 3xTg and Thy1-ApoE4/C/EBPβ Tg mice at 2-, 6- and 12-month age. Scale bar 500 μm for top panels, 20 μm for bottom panels. **D** and **E**. Co-staining of Aβ plaque with ThS **D** and Aβ plaque with AV45 **E** in brain of 3xTg and Thy1-ApoE4/C/EBPβ Tg mice. Scale bar, 20 μm. **F** Levels of Aβ42, Aβ40 and ratio of Aβ42/Aβ40 in the CSF of 3xTg and Thy1-ApoE4/C/EBPβ Tg mice. (*n* = 3, *p < 0.05, **p < 0.01, ***p < 0.001).

PET imaging can quantitatively map amyloid accumulation in living amyloid precursor protein (APP) transgenic mice [40]. To side-by-side compare Aβ aggregation pathology in live brains of the AD mouse models, we used ^18^F-florbetapir (AV45) as a PET tracer to probe the Aβ plaque deposition. Noticeably, 3xTg and Thy1-ApoE4/C/EBPβ Tg mice exhibited significantly Aβ PET signals elevation at 6 months of age, which were further augmented at 12 months old as compared to control mice. The images from coronal, transverse and sagittal views demonstrated the age-dependent PET signals escalation (Fig. 3A, B). IF staining with anti-Aβ 4G8 antibody showed that Aβ aggregates were demonstrable at 2 months old and the accumulates gradually grew and the sizes became larger and larger in both 3xTg and Thy1-ApoE4/C/EBPβ Tg mice from 6 to 12 months of age (Fig. 3C). Anti-Aβ and Thioflavin S (ThS) co-staining revealed that the aggregated Aβ in the brain sections from 3xTg and Thy1-ApoE4/C/EBPβ Tg mice were pathological β-sheet inclusions (Fig. 3D). Anti-Aβ and AV45 co-staining also validated the Aβ proteinaceous aggregates were similar folding conformation, corroborating with the in vivo PET imaging. Markedly, human Aβ plaques in 3xTg appeared fragmented and loose, whereas mouse Aβ plaques were tighter and slightly smaller (Fig. 3E). Next, we quantitatively analyzed human and mouse Aβ42/40 ratios in the CSF from 3xTg and Thy1-ApoE4/C/EBPβ Tg mice, respectively and found that Aβ42/40 ratios were age dependently decreased in both 3xTg and Thy1-ApoE4/C/EBPβ Tg mice (Fig. 3F). Moreover, coronal section and sagittal section staining with anti-Aβ revealed extensive Aβ plaque deposits in the forebrain with cortex most abundant (Supplementary Fig. 3A–C).

Mouse Aβ has been questioned to aggregate into pathological fibrils, though extensive previous studies support that mouse Aβ and mouse Tau undeniably aggregate into amyloid deposits [41–43], mimicking the pathological features in human AD patient brains. To further characterize accumulated Aβ structures from the two AD mouse models, we isolated the Aβ inclusions from the brain and performed electron microscope (EM) analysis. They both exhibited similar fibrillary structures (Supplementary Fig. 4A, B). They displayed smeared bands with large molecular weights on the IB SDS-PAGE gels, indicating the aggregated molecules (Supplementary Fig. 4C). Quantification showed mouse Aβ42 and 40 levels age-dependent elevation and Aβ40 concentrations in Thy1-ApoE4/C/EBPβ Tg mice were significantly higher than control mice starting at 6 months old, and further augmented at 12 months old, whereas mouse Aβ42 levels in Thy1-ApoE4/C/EBPβ Tg mice only significantly higher than control mice at 12 month old. By contrast, human Aβ42 from 3xTg were gradually increased at both 6 and 12 months old, and both of which were significantly higher than 2 months old. On the other hand, human Aβ40 at 12 months old was higher than 2 months old (Supplementary Fig. 4D). In the insoluble fractions, we found that both mouse Aβ 42 and Aβ40 levels from Thy1-ApoE4/C/EBPβ mice were much more abundant at 6 and 12 months than control mice, and human Aβ42 and 40 in 3xTg mice at 6 and 12 months old were significantly higher than 2 months old (Supplementary Fig. 4E). Aggregated Aβ fibrils are neurotoxic. To compare whether isolated Aβ aggregated from these mice display similar actions, we prepared primary neuronal cultures and incubated with Aβ aggregated (2 μg) from the 2 AD mouse models, and IF co-staining showed that Aβ induced AT8-positive p-Tau signaling in the treated neurons. They also elicited extensive neuronal apoptosis as revealed by TUNEL staining. Noticeably, they triggered AEP activation, and Tau N368 fragmentation in the treated neurons (Supplementary Fig. 4F–H), supporting that purified Aβ aggregates are neurotoxic and provoking Tau proteolytic cleavage and hyperphosphorylation in neurons.

### Mouse-derived Aβ aggregates spread in the brain of APP/PS mice

**Fig. 4:**
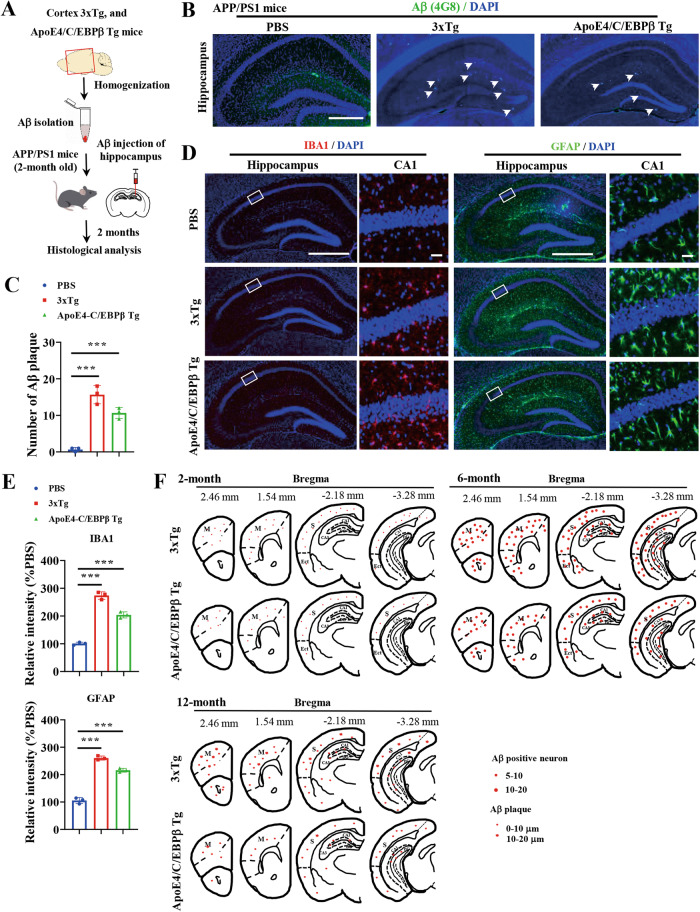
Aβ aggregates extracted from 3xTg and Thy1-ApoE4/C/EBPβ Tg mice induce endogenous amyloid-β accumulation in APP/PS1 mice. **A** Diagram showing in vivo function assay of Aβ aggregates isolated from 3xTg and Thy1-ApoE4/C/EBPβ Tg mice. Aβ aggregates were extracted from cortex and injected into hippocampus of APP/PS1 mouse. After 2 months of injection, the mice were sacrificed for histological analysis in brain. **B** Representative of Aβ plaque immunostaining images in the hippocampus of APP/PS1 mice inoculated with Aβ aggregates isolated from 3xTg and Thy1-ApoE4/C/EBPβ Tg mice. Scale bar 500 μm. **C** Relative quantification of Aβ plaque number in **B**. **D** Representative of IBA1 (left two panel) and GFAP (right two panel) immunostaining images in hippocampus of APP/PS1 mice inoculation with Aβ aggregates. Scale bar 500 μm for column 1 and 3, 20 μm for column 2 and 4. **E** Relative quantification of IBA1 and GFAP fluoresce intensity in **D**. **F** Schematic drawings representing the distribution of Aβ positive neurons and Aβ plaque in 3xTg and Thy1/ApoE3/C/EBPβ transgenic mice at the age of 2-, 6- and 12-month of age. Note the age-dependent increase in the number of Aβ (4G8) positive neurons in the cortex and hippocampus. CA1: CA1 hippocampal subfield, CA3 CA3 hippocampal subfield, DG dentate gyrus, Ect ectorhinal cortex, M motor cortex, S sensory cortex, Small dots = 5–10 positive neurons (2-month age), medium-size dots = 20–40 positive neurons (6-month age), star = Aβ plaque at different size (12-month age).

Intracerebral injection of dilute, Aβ-containing brain extracts from AD patients or human APP transgenic mice induces cerebral β-amyloidosis and associated pathology in APP transgenic mice in a time- and concentration-dependent manner [44]. To assess whether mouse Aβ aggregates can also spread in 2-month old APP/PS1 host mouse brain, we prepared the Aβ extracts from 3xTg and Thy1-ApoE4/C/EBPβ mouse brains and injected into the hippocampus. In 2 months, we found induced Aβ deposits primarily in the injected hippocampus and most induced Aβ deposits were diffuse (Fig. 4A–C). Noticeably, Iba-1 and GFAP IF signals were strongly increased in the hippocampus in host mouse brains after Aβ inoculation as compared to vehicle control, indicating pronounced microglia activation and astrogliosis (Fig. 4D, E), consistent with previous observations [44]. Next, we monitored Aβ propagation in 3xTg and Thy1-ApoE4/C/EBPβ mouse brains at 2, 6 and 12 months old, respectively, and found that intraneuronal Aβ signals were primarily enriched in the forebrain and frontal cortex at 2 months, and the Aβ-positive neurons gradually increased in the cortex and hippocampus at 6 months, and different Aβ plaque deposits were demonstrable in these brain regions at 12 months of age. The heat map is shown in Fig. 4F. Therefore, human Aβ in 3xTg and mouse Aβ in Thy1-ApoE4/C/EBPβ mice are originated from the intraneuronal accumulation to extra-cellular plaque deposition in the forebrain, mimicking Aβ propagation in AD patients.

### Age-dependent Tau PET signals in the brains of 3xTg and Thy1-ApoE4/C/EBPβ Tg mice

**Fig. 5:**
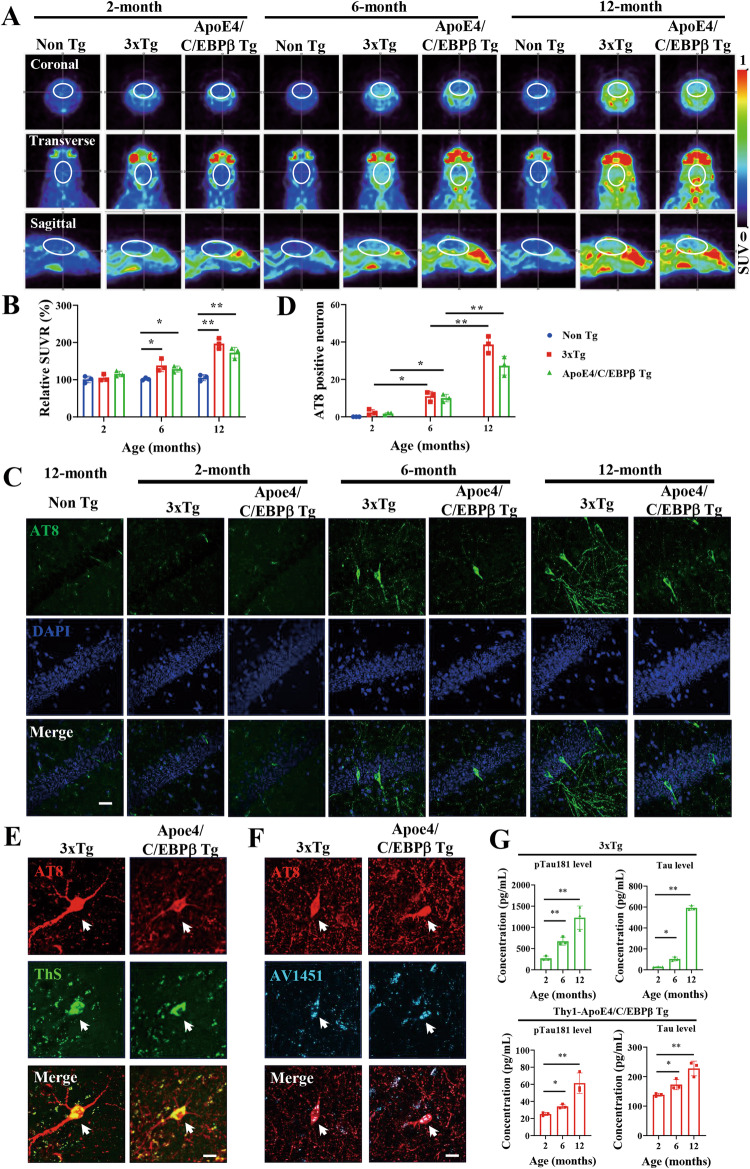
Age-dependent increase of Tau pathology in 3xTg and Thy1-ApoE4/C/EBPβ Tg mice. **A** Representative Tau PET images showing the Tau deposition in the brains of 3xTg and Thy1-ApoE4/C/EBPβ Tg mice at 2-, 6- and 12-month of age. 18F-AV1451 was used as a PET tracer of Tau aggregates. **B** Relative quantification of Tau PET standard uptake ratio (SUVR) in **A** (*n* = 3 mice, **p < 0.01, compared with Non Tg). **C** Representative of AT8 immunostaining images in the hippocampus of 3xTg and Thy1-ApoE4/C/EBPβ Tg mice at 2-, 6- and 12-month of age. The Non Tg mice have no AT8 signals at all ages. Scale bar, 20 μm. **D** Relative quantification of AT8 positive neurons in **C**. (*n* = 3 mice, *p < 0.05, **p < 0.01). **E** Co-staining of AT8 with ThS in the brains of 3xTg and Thy1-ApoE4/C/EBPβ Tg mice. Scale bar 20 μm. **F** Co-staining of AT8 with AV1451 in the brains of 3xTg and Thy1-ApoE4/C/EBPβ Tg mice. Scale bar 20 μm. **G** Levels of pTau181 and total Tau in the CSF of 3xTg (Top panels) and Thy1-ApoE4/C/EBPβ Tg mice (Bottom panels). (*n* = 3 mice, *p < 0.05, **p < 0.01).

To compare the Tau-laden neurofibrillary tangle pathology between 3xTg and Thy1-ApoE4/C/EBPβ mice, we conducted neuroimaging to examine Tau pathology in live animals using ^18^F-AV1451, as a PET tracer for Tau aggregates, and found the radioactive intensities progressively increased in coronal, transverse and sagittal views in both AD mouse models as compared to control mice. Quantification showed that the relative SUVR (standard uptake value ratio) significantly elevated versus control mice at 6 months of age, which was further augmented at 12 months old in both AD mouse models (Fig. 5A, B). IF staining revealed that AT8 fluorescent activities in AD mouse models grew with aging and Tau hyperphosphorylation signals were much higher at 6 and 12 months of age compared to 2 months old 3xTg and Thy1-ApoE4/C/EBPβ mice (Fig. 5C, D). IF co-staining validated that AT8 positive Tau aggregates were ThS positive fibrillary tangles, which were further confirmed by AV1451 cold PET tracer staining (Fig. 5E, F). In addition, we quantitatively analyzed total Tau and p-Tau 181 in the CSF from 3xTg and Thy1-ApoE4/C/EBPβ Tg mice, and found that both total Tau and p-Tau 181 increased in an age dependent manner (Fig. 5G). Therefore, mouse Tau in Thy1-ApoE4/C/EBPβ mice also mimic human Tau in 3xTg aggregate into neurofibrillary tangles with aging.

### Mouse-derived Tau aggregates are infectious and neurotoxic

**Fig. 6:**
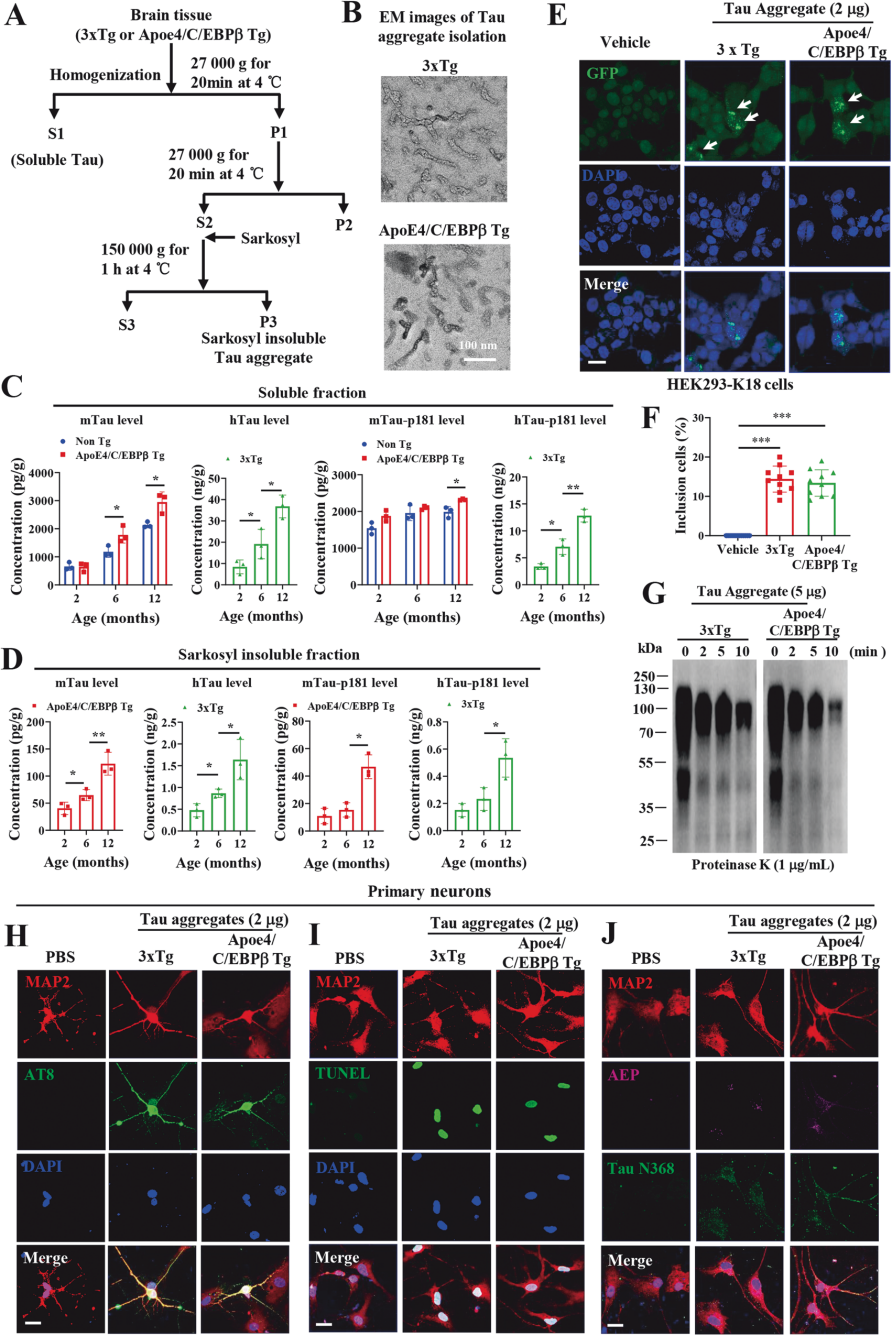
Characterization of Tau aggregates in 3xTg and Thy1-ApoE4/C/EBPβ Tg mice. **A** Diagram showing the isolation of soluble and insoluble Tau in the brain tissues of 3xTg and Thy1-ApoE4/C/EBPβ Tg mice. **B** Representative electron microscope images of Tau insoluble fraction extracted from the brains of 3xTg and Thy1-ApoE4/C/EBPβ Tg mice. Scale bar 100 nm. **C** ELISA assay of soluble Tau levels in the brains of 3xTg and Thy1-ApoE4/C/EBPβ Tg mice at different ages. (*n* = 3 mice, *p < 0.05, **p < 0.01). **D** ELISA assay of insoluble Tau levels in the brains of 3xTg and Thy1-ApoE4/C/EBPβ Tg mice at different ages. (*n* = 3 mice, *p < 0.05, **p < 0.01). Tau aggregates (2 μg) from the brains of 3xTg and Thy1-ApoE4/C/EBPβ Tg mice were transduced into HEK293-K18 cells stably expressing GFP-tagged Tau RD (repeat domain), After 24 h of transduction, the insoluble Tau inclusions in cells were imaged **E** and quantified **F** under microscopy. (***p < 0.01 as compared with vehicle). **G** Tau aggregates (5 μg) extracted from the brains of 3xTg and Thy1-ApoE4/C/EBPβ Tg mice were digested with Protease K (1 μg/ml) at different time points, and then immunoblotted with Tau antibody. Representative immunostaining images of AT8 **H** and TUNEL **I**, AEP and Tau N368 **J** in primary rat neurons treated with Tau aggregates (2 μg) extracted from the brains of 3xTg and Thy1-ApoE4/C/EBPβ Tg mice. Scale bar 20 μm.

Mouse Taus show 89% identities with human Taus, and they were questioned to aggregate into pathological fibrils. Previous study supports that mouse Tau indisputably aggregate into amyloid deposits and NFT [45], mimicking the pathological features in human AD patient brains. To interrogate whether mouse Tau in Thy1-ApoE4/C/EBPβ mice demonstrates similar paired helical fibrils, we prepared Tau aggregates from both 3xTg and Thy1-ApoE4/C/EBPβ mouse brains and EM images revealed that both human and mouse Tau aggregates displayed similar fibrils structures (Fig. 6A, B). Quantification indicated that mouse Tau (mTau) levels in soluble fractions exhibited age-dependent escalation with more abundant mTau in Thy1-ApoE4/C/EBPβ mice than control mice at 6 and 12 months of age, and p-Tau 181 levels in Thy1-ApoE4/C/EBPβ mice were significantly higher in control mice. Both total hTau and p-Tau181 levels in 3xTg also displayed augmentation with aging (Fig. 6C). In insoluble fractions, Tau and p-Tau levels for both human and mouse progressively escalated from 2 to 6 and to 12 months old (Fig. 6D). It is worth noting that these Tau aggregates elicited Tau accumulation in human Tau stable transfected HEK293-K18 cells, indicating that mouse Tau simulates human Tau in triggering Tau aggregation in intact cells (Fig. 6E, F). They both exhibited smear aggregation in SDS-PAGE gels and demonstrated a time-dependent degradation by proteinase K (Fig. 6G). Moreover, they also induced endogenous Tau hyperphosphorylation and neuronal apoptosis in primary neuronal cultures. As expected, both of them stimulated AEP activation and Tau N368 fragmentation as well (Fig. 6H-J), underscoring that mouse Tau like human Tau aggregates are infectious and neurotoxic.

IF co-staining studies showed that Tau N368 and AT8 fluorescent intensities elevated with aging in the hippocampus of both 3xTg and Thy1-ApoE4/C/EBPβ mouse brains (Supplementary Fig. 5A, B). T22, a specific antibody for aggregated Tau tangles, and Tau N368 co-staining also supported that AEP-truncated Tau N368 co-localized with T22 signals that were gradually augmented in an age-dependent manner in both 3xTg and Thy1-ApoE4/C/EBPβ mice (Supplementary Fig. 5C, D). Thus, AEP is progressively activated in these AD mouse brains, cleaving Tau N368 that elicits Tau hyperphosphorylation and aggregation, consistent with our previous findings [27]. Tau lesion in AD originates in the locus coeruleus (LC) neurons, which subsequently spread to entorhinal cortex (EC), and then to hippocampus (HC) [46]. To explore whether Tau pathology propagate in mouse models in a similar pathway, we monitored Tau pathology spreading routes. LC neurons were labeled with anti-DBH (Dopamine β-hydroxylase), a marker for norepinephrine neurons. IF co-staining revealed AT8 and T22 fluorescent signals were detectable at 2 months old, and these signals augmented with aging in the LC regions in both AD mouse models. At 6 months old, AT8 and T22 signals were demonstrable in EC and HC (Supplementary Fig. 6). Human Tau pathologies appeared stronger than mouse counterpart, which might be due to human Tau is Tau P301S mutant in 3xTg, whereas mouse Tau in Thy1-ApoE4/C/EBPβ mice is wild-type.

### Mouse-derived Tau aggregates spread in the brains of 3xTg and Thy1-ApoE4/C/EBPβ Tg mice

**Fig. 7:**
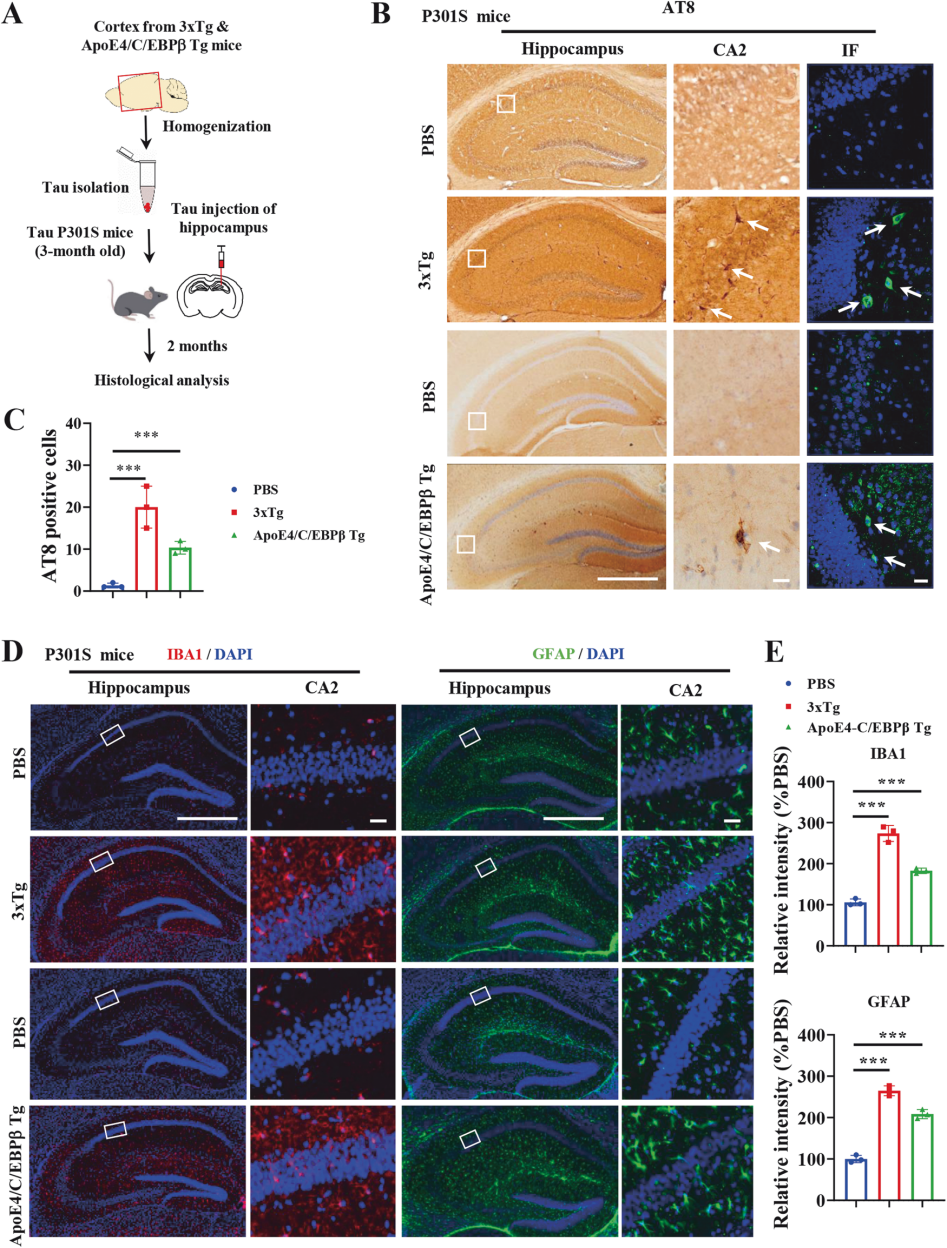
In vivo functions of Tau aggregates extracted from 3xTg and Thy1-ApoE4/C/EBPβ Tg mice. **A** Diagram showing in vivo function assay of Tau aggregates isolated from 3xTg and Thy1-ApoE4/C/EBPβ Tg mice. **B** Representative of AT8 immunostaining images in the hippocampus of Tau P301S mice inoculation with Tau aggregates isolated from 3xTg and Thy1-ApoE4/C/EBPβ Tg mice. Scale bar 500 μm for left panels, 20 μm for right panels. **C** Relative quantification of AT8 positive neurons in **B**. (*n* = 3 mice, *p < 0.05, **p < 0.01). **D** Representative of IBA1 (left two panels) and GFAP (right two panels) immunostaining images in the hippocampus of Tau P301S mice inoculation with Tau aggregates isolated from 3xTg and Thy1-ApoE4/C/EBPβ Tg mice. **E** Quantification of IBA1 and GFAP fluoresce intensity in **D**. (***p < 0.01 as compared with PBS).

To investigate whether mouse Tau aggregates could also spread in live animal’s brain, we isolated Tau aggregates from the cortex from both 3xTg and Thy1-ApoE4/C/EBPβ mice, and injected into the hippocampus of 3-month old Tau P301S mice. In 2 months, we found robust AT8 signals appeared in a lot of hippocampal neurons validated by both IF staining and immunohistochemistry (IHC) staining (Fig. 7A–C). Again, we also observed extensive Iba-1 and GFAP fluorescent activities in the hippocampus regions, suggesting that inoculated Tau fibrils incurs prominent microglia activation and astrogliosis (Fig. 7D, E).

Neuroinflammation is one of the key hallmarks in AD pathology. Quantitative RT-PCR (qRT-PCR) analysis showed that both IL-6 and TNFα mRNA levels were significantly increased in both 3xTg and Thy1-ApoE4/C/EBPβ mice compared to control mice at 6 and 12 months old. ELISA quantification of these inflammatory cytokines in the brain lysates revealed similar augmentation patterns (Supplementary Fig. 7A, B). qRT-PCR of GFAP and Iba-1 mRNA also showed that both concentrations increased with aging with 6 and 12 months of ages significantly higher in 3xTg and Thy1-ApoE4/C/EBPβ mice compared to control mice (Supplementary Fig. 7C). IF staining of the brain sections confirmed the qRT-PCR results (Supplementary Fig. 7D, E). Therefore, Thy1-ApoE4/C/EBPβ mice exhibit widespread neuroinflammation like 3xTg mice, mirroring what observed in human AD patient brains.

### ApoE4 promotes mouse Aβ polypeptide aggregation in vitro

Mouse Aβ42 possesses 3 different amino acids from human counterpart. Compared to human Aβ42 polypeptide, mouse Aβ42 are poorly aggregated into fibrils in vitro [47, 48]. To explore why mouse Aβ polypeptide could aggregate into plaques in Thy1-ApoE4/C/EBPβ mice, we perform in vitro aggregation assay by shaking the peptides at 37 ^o^C in vitro. Thioflavin T (ThT) fluorescent kinetic curves showed that mouse Aβ42 failed to aggregate, in contrast human Aβ42 aggregates in a time-dependent manner. Noticeably, mouse Aβ42 started to aggregated in the presence of recombinant ApoE proteins with ApoE4 stronger than ApoE3 (Supplementary Fig. 8A). EM analysis validated mouse Aβ42 formed extensive fibrils in the presence of ApoE4, whose morphologies appeared different form human counterpart (Supplementary Fig. 8B). IB analysis with these samples demonstrated that massive human Aβ42 oligomers, which were also found in mouse Aβ42/ApoE4 group. By contrast, the oligomer amount was decreased in ApoE3 (Supplementary Fig. 8C). Interestingly, these Aβ42 oligomers also triggered significant cell death in SH-SY5Y cells, which correlated with their effects in activating AEP (Supplementary Fig. 8D, E). IB showed that both human Aβ42 and mouse Aβ42 oligomers strongly activates C/EBPβ/AEP signaling (Supplementary Fig. 8F, G). As expected, these oligomers also strongly triggered microglia cell activation, leading to inflammatory cytokines including TNFα and IL-6 expression and secretion (Supplementary Fig. 8H, I). Hence, mouse Aβ42 in Thy1-ApoE4/C/EBPβ Tg mice are aggregated into amyloid plaques in the presence of ApoE4, simulating the pathological functions of human Aβ42.

## Discussion

In the current study, we utilized the clinical diagnostic criteria for AD patients to fully characterize Thy1-ApoE4/C/EBPβ Tg mice, and compared with genetic 3xTg AD mouse model side-by-side. We show that C/EBPβ/AEP signaling is activated in both AD mouse models, associated with APP N585 and Tau N368 fragmentation, which correlates with potent AEP enzymatic activities. Consequently, Aβ PET and Tau PET validate abundant Aβ plaques and Tau tangles deposited in the brains, which become apparent at 6 months old in both AD models. The Aβ and Tau pathologies temporally couple to C/EBPβ/AEP biochemical events, underscoring that APP and Tau fragmentation by AEP is accountable for the amyloid and Tau pathologies. Moreover, the mouse Aβ and Tau aggregates isolated from Thy1-ApoE4/C/EBPβ Tg mice are structurally similar to human counterparts purified from 3xTg mice. In addition, they are all infectious and neurotoxic. Inoculation of these isolated pathological fibrils into the hippocampus of APP/PS1 or Tau P301S host mice spread the Aβ and Tau pathologies to other regions, associated with widespread neuroinflammation. In alignment with human fibrils propagation, both mouse Aβ and Tau pathologies spread in a similar manner as in human AD patient. Specifically, Aβ pathology starts from the forebrain cortex to propagate to the whole cortex, then to the midbrain, and subsequently to the brain stem; by contrast, Tau lesion originates from the LC neurons in the brain stem to EC then to HC, finally spread to the whole cortex [46]. Our analysis shows that mouse Aβ and Tau reveal the similar propagation routes in Thy1-ApoE4/C/EBPβ Tg mice to human counterparts in 3xTg AD mice. Accordingly, MRI demonstrates that both animal models exhibit the brain volume reduction at the similar time points. Consequently, the spatial memory and object memory defects take place at 6-month old and 12-month old in both models, respectively. Therefore, Thy1-ApoE4/C/EBPβ model mimics the development of sporadic AD pathologies more closely, and this model is better suited for studying the multifactorial and non-genetic aspects of AD pathogenesis. In contrast, the commonly used mouse models, including 3xTg, 5xFAD and Tau P301S, carry several mutant genes associated with familial AD, representing the rare familial form of the disease. While valuable for studying the genetic aspects of AD, these models do not fully capture the complexity of sporadic AD.

MMSE, MRI, CSF Aβ42 / Aβ40 ratio, p-Tau and total Tau levels, Aβ PET and Tau PET signals are standard clinical diagnosis protocols for AD patients. We have shown that CSF mouse Aβ42/Aβ40 ratios reduce in Thy1-ApoE4/C/EBPβ Tg mice, similar to human Aβ42/Aβ40 ratios decrease in 3xTg mice, supporting that mouse Aβ42 is gradually aggregated and accumulated in the brain. These findings are in alignment with progressively augmented Aβ PET signals in Thy1-ApoE4/C/EBPβ Tg mice (Fig. 3). Moreover, our previous results showed that CSF Tau N368/Tau ratios in AD patients better reflect the individual cognitive deficits and Tau pathologies than p-Tau 181 and p-Tau217 levels [49, 50]. Consistently, we observed age-dependent Tau N368 fluorescent signals escalation in both 3xTg and Thy1-ApoE4/C/EBPβ Tg mice, tightly correlating with gradual elevation of AT8 and T22 activities (Supplementary Fig. 5). Because Tau N368 is prior to Tau hyperphosphorylation and NFT pathology [27]. Conceivably, Tau N368 is a promising biomarker for early diagnosis of AD.

Synaptic dysfunction is among the best correlates for the memory and cognitive changes that characterize AD [3, 4]. The dendritic arborization and spines are distinctly attenuated in the hippocampal CA1 neurons in 9 months old Thy1-ApoE4/C/EBPβ Tg mice versus the WT littermates of the same age. In addition, EM study shows that the synapses are evidently reduced in Thy1-ApoE4/C/EBPβ Tg mice with aging. Consistently, the electrophysiology analysis demonstrates that synaptic plasticity (long-term potentiation, LTP) in Thy1-ApoE4/C/EBPβ Tg mice is diminished in an age-dependent manner [34]. Hence, Thy1-ApoE4/C/EBPβ Tg mice display synaptic plasticity defects with impaired LTP, correlating with wide-spread synapse loss and reduced dendritic spines. 3xTg mice reveal deficits in long-term synaptic plasticity that correlates with the accumulation of intraneuronal Aβ, suggesting a pathogenic role in sabotaging synaptic plasticity by intraneuronal Aβ [36]. LTP is severely impaired in the 6-month-old 3×Tg AD mice. The intraneuronal accumulation of Aβ underlies the observed synaptic dysfunction. We found that extensive Aβ-positive neurons and abundant Aβ plaques deposited in the hippocampus of 6-month old 3xTg and Thy1-ApoE4/C/EBPβ mice (Figs. 3, 4).

In order to address whether mouse Aβ or Tau could aggregate into pathological fibrils, we provide a plethora of evidence showing that ThS and cold PET tracers AV45 and AV1451 form fluorescent complex in the β-sheet enriched fibrils (Figs. 3, 5), which are corroborated by EM analysis (Fig. S4, Fig. 6). In addition, these isolated amyloid and Tau aggregates are infectious and neurotoxic, similar to their human counterparts purified from 3xTg mice. The Aβ and Tau proteins misfold, self-assemble, and propagate by an endogenous mechanism closely resembling the seeded aggregation and spread of the prion proteins [51–53]. Aβ plaques disrupt circuits and neighboring brain cells [54, 55], but Aβ also forms small, soluble, oligomeric assemblies that impair the function of neurons and glia [56, 57]. Inoculation of the amyloid or Tau aggregates into APP/PS1 or Tau P301S mice elicits Aβ and Tau pathology spreading (Figs. 4, 6, 7). These results are in agreement with extensive previous studies showing that mouse Aβ and mouse Tau undeniably aggregate into amyloid deposits and NFT [41–43, 45], mimicking the pathological features in human AD patient brains. With the aim of supporting that mouse Aβ and mouse Tau indeed aggregate into amyloid deposits and NFT, we employed various approaches to confirm their identities including immunoblotting and proteomics from the insoluble fractions from mouse brains, anti-Aβ or anti-AT8 co-staining with ThS, Gyllas-Braak Silver staining and immuno-EM [34]. Hence, mouse Aβ and mouse Tau can form pathological amyloid deposits and NFT as human counterparts in AD patients.

Previous studies show that mouse Aβ is less prone to aggregate into pathological fibrils [47, 48]. In aging APP (V717F ^+/-^) transgenic mice expressing mouse ApoE, no ApoE, or human ApoE2, ApoE3, or ApoE4, Holtzman et al. [58] demonstrate that ApoE facilitates, but is not required for, Aβ fibril formation in vivo. Human ApoE isoforms markedly delays Aβ deposition relative to mouse ApoE, with ApoE2 (and ApoE3 to a lesser extent) having a prolonged ability to prevent Aβ from converting into fibrillar forms [59]. In this study, it is human Aβ in vivo aggregation is analyzed. However, we show mouse Aβ alone is unable to aggregated, fitting with previous reports. On the contrast, human ApoEs prominently stimulate mouse Aβ fibrillization in vitro with ApoE4 stronger than ApoE3. Furthermore, the in vitro mouse fibrils are neurotoxic and trigger C/EBPβ/AEP signaling activation, in addition to incurring microglia activation and inflammatory cytokines secretion (Supplementary Fig. 8). These findings shed insights into the molecular mechanism why endogenous mouse Aβ and Tau machinery in Thy1-ApoE4/C/EBPβ Tg mice could tempo-spatially reconstitute salient features of AD pathologies in the absence of any APP or PS1/2 mutation.

AD patient brains display cerebral atrophy and white matter changes by antemortem MRI reflect underlying neuropathology. Brain volume loss correlates more strongly with tangles than with any other pathological finding [38]. MRI assay included assessment of hippocampal structural integrity [39]. Here, we show that Thy1-ApoE4/C/EBPβ Tg and 3xTg mice exhibit age-dependent brain atrophy and hippocampal and cortical volume reduction validated by MRI (Fig. 2), which are consistent with temporal neuronal cell death in both mice (Supplementary Fig. 2). We find that both 3xTg and Thy1-ApoE4/C/EBPβ Tg AD mice progressively develop Aβ and Tau pathology, with a temporal- and regional-specific profile that closely mimics their development in the human AD brain (Figs. 3, 5). Aβ deposits initiate in the cortex and progress to the hippocampus with aging, whereas Tau pathology is originated from the LC to EC to HC and then progresses to the cortex (Supplementary Fig. 6).

Recent anti-Aβ monoclonal antibody trials reveal that removing aggregated Aβ from the brains of symptomatic patients slows AD progression. However, the achieved clinical benefit has been modest, suggesting the need for both a deeper understanding of disease mechanisms and the importance of intervening earlier [60]. C/EBPβ is a transcription factor for APP, MAPT and BACE1 [34], which are subsequently cleaved by AEP into APP N585 and Tau N368 and BACE1 N294, respectively, stimulating Aβ and p-Tau pathologies [27, 28, 37]. In addition, it also mediates ApoE mRNA transcription as well [31] and ApoE4 feeds back and activates C/EBPβ with 27-hyroxycholesterol, driving AD pathology [32]. In our most recent study, we find that neuronal but not glial ApoE4 is essential for jointly activating this pathway with FSH [61]. Our study further confirms the toxic role of neuronal ApoE in AD pathogenesis [34, 62, 63]. Consequently, neuronal ApoE4 stimulates C/EBPβ activation in Thy1-ApoE4/C/EBPβ transgenic mice, promoting AD pathologies via mouse machinery. Together, our thorough characterization of the double transgenic mice supports the animal acts as a sporadic AD model and reproduces both amyloid and Tau pathologies in a regional and temporal pattern analogous to AD patients, which makes it very valuable for longitudinal studies. Conceivably, this sporadic AD mouse model will provide an innovative tool for therapeutic agents’ treatment investigation.

## Materials and methods

### Mice and reagents

#### Mice

The 3xTg mice, Tau P301S (line PS19) and APP/PS1 mice were originally purchased from the Jackson Laboratory (3xTg, catalog no. 008169; Tau P301S, catalog no. 008169; APP/PS1, catalog no. 005864). The Thy1-ApoE4/C/EBPβ transgenic mice were generated and maintained as described in our previous work [34]. The mice carrying one copy of ApoE4 and human C/EBPβ genes, i.e., ApoE4/C/EBPβ Tg, were used for the study unless otherwise stated. All the mice were bred in specific pathogen–free facilities, with 12-h light/12-h dark cycle and free access to water and food, at Shenzhen Institute of Advanced Technology (SIAT), Chinese Academy of Sciences (CAS). Both male and female mice, with the age of 2, 6 and 12 months, were interchangeably used for experiments. All protocols involving experimental animal were approved by the institutional animal care and use committee at the SIAT.

### Antibodies and reagents

All antibodies used in this work are listed as followed: anti-amyloid β (Aβ, clone, 4G8, BioLegend, 800701), anti-NeuN (Sigma Aldrich, MAB377), anti-Synaptophysin (Proteintech, 17785-1-AP), anti-PSD95 (Cell signaling technology, #3409), anti-synapsin 1 (Cell signaling technology, #5297), anti-APP (Sigma-Aldrich, MAB348), anti-APP N585 (Ye lab), anti-APP C586 (Ye lab), anti-Tau N368 (Ye lab), anti-GFAP (Invitrogen, PA516291), anti-Iba1 (Invitrogen, PA5-18039), Anti-Tau 5 (Sigma Aldrich, MAB361), anti-active AEP antibody (Cell signaling technology, #93627), MAP2 (Proteintech, 17490-1), Anti-AT8 (phospho-Tau at Ser 202, Thr205, Thermo Fisher, MN1020), Anti-C/EBPβ (Santa Cruz, sc-7962), Anti-p-C/EBPβ (Cell Signaling Technology, #3084) and anti-GAPDH (ProteinTech, 60004-1), anti-T22 (Millipore, ABN454). Alexa Fluor 594, 488 and 647 conjugated secondary antibodies were purchased from Jackson ImmunoResearch.

AEP substrate Z-Ala-Ala-Asn-AMC (Bachem, 4033201), DAPI (Sigma Aldrich, D9542), Mouse and Rabbit Specific HRP/DAB IHC Detection Kit (Abcam, ab236466). Human Aβ40, Aβ42, Tau, pTau181 and ELISA kits, pro-inflammatory cytokines TNF-α, IL-6 ELISA kits were purchased from Invitrogen. Mouse Aβ40, Aβ42, Tau, pTau181 and ELISA kits, pro-inflammatory cytokines IL-1β, TNF-α, IL-6 ELISA kits were purchased from a commercial company (Aimeng Youning, Shanghai, China). Cold tracer of Aβ (AV-45, HY-129650) and Tau (AV-1451, HY-101184) were purchased from Med Chem Express (USA). Human and mouse Aβ 42 peptide were purchased from ChinaPeptides (China).

### Behavioral tests

#### Morris water maze

Mice were trained in a round tub filled with water in an environment surrounded by extra maze cues. Each mouse was trained 3 trails/day for 5 consecutive days with a 15-min intertrial interval. The maximum trial time was 60 s, and if the mouse did not reach the platform in the allotted time, they were manually guided to do it. Following the 5 days task training, a probe trial was given, during which time the platform was removed. All trials were analyzed for latency using EthoVision XT-Video tracking software (Noldus Information Technology Co., Ltd).

#### Novel object recognition test (NORT)

Mice subjected to NORT underwent three phases: habituation, familiarization and discrimination. In the habituation phase, each mouse was placed in a square open field (40 × 40 × 40 cm) individually for 10 min per day for three consecutive days. Then the animals entered into the familiarization phase. In this phase, each mouse was allowed to explore in the open field with two identical objects located in opposite and equidistant positions for 10 min. Exploration  was  defined  as  sniffing  or  touching  the  objects  with  the  nose  and/or  forepaws  when  the  nose  was  in  contact  with  or  directed  at  the  object at a distance of ≤ 1.5 cm.  After a three-hour retention interval, the mouse returned to the open field, with one of the familiar objects replaced by a novel object. For the discrimination phase, each mouse was allowed to explore for 10 min and the time for exploring each object was recorded. Mice touching an object or facing an object within 2 cm around the object were taken as measure of object exploration behavior. To eliminate olfactory cues, the objects and field were cleaned with 75% ethanol between each trial. The exploring index was determined as: (exploration time for the novel - exploration time for the familiar object) / (total exploration time during the test session) × 100%.

### Brain volume quantification

#### MRI acquisition

MRI experiments were performed on a United Imaging uMR 9.4 T scanner with a standard cross coil set-up using a volume coil for excitation and quadrature mouse surface coil for signal detection (UNITED IMAGING, Shanghai, China). Mice were kept anesthetized by isoflurane, and T2-weighted images were acquired in coronal planes using a rapid acquisition with relaxation enhancement sequence with the following parameters: TR = 3500 ms; TE = 47 ms; pixel size of 0.078 mm × 0.078 mm and slice thickness of 0.2 mm without spacing between slices. Regional volumes were determined using U_VIEWER software (UNITED IMAGING, Shanghai, China).

Nissl staining was further used for brain volume quantification as described in our previous work [34]. In brief, mice hemibrains were cut coronally at 30 μm, and all sections were collected. Brain sections were mounted on microscope slides (Fisher Scientific) in an anterior-to-posterior order, starting from the section where the target structure first becomes visible (first section) to the section where target structure just disappears (last section). Mounted brain sections were dried at room temperature and stained with Cresyl violet (Nissl staining). Sections were stained in 0.1% Cresyl violet solution (Abcam) and mounted. Images were acquired with an Olympus VS120 virtual microscopy slide scanning system. The volume of ventricle and hippocampus were estimated using Image J.

### Small animal PET

Mice were anesthetized following a standardized protocol for Radiochemistry, acquisition, and post processing. Inhalation anesthesia with isoflurane was used throughout the experiments, and PET images were recorded on a high-resolution small animal PET scanning device (microPET) with a spatial resolution of 1.0 mm. Brain emission scans were obtained in volumetric mode for the 20 min after an intravenous injection of 10–15 MBq of 18F-Florbetapir (AV45) or 18F-Flortaucipir (AV1451) in approximately 100 μL of saline into the vein of tail. The PET images were reconstructed by using ordered-subset expectation maximization (OSEM) with 16 subsets and 5 iterations. Target–to–reference tissue (the cerebellum) standard uptake value ratio (SUVR) was calculated from a standardized target volume of interest, and was analyzed by Amide 1.0.4-1(San Diego, CA 92101) for scaling of 18F-AV45 or 18F-AV1451 data.

### CSF and serum collection

Collection of CSF from the cisterna magna was carried out as previously described [64]. In brief, the mice were deeply anesthetized by 1% pentobarbital sodium and then placed in a stereotaxic device. The skin of the neck was shaved, and the surgical site was swabbed with 10% povidone iodine, followed by 70% ethanol. A sagittal incision of the skin was made inferior to the occiput, and the subcutaneous tissue and muscles were separated under the dissection microscope. After the CSF space was visible, a glass capillary tube was inserted into the cisterna magna, and then the CSF was collected. Samples showing signs of blood contamination were centrifuged at 2000 g for 10 min at room temperature. For serum extraction, the whole-blood samples were collected from the mice and then centrifuged at 3000 g for 10 min at 4 °C. All the CSF and serum samples were stored at −80 °C until use.

### SiMoA assay of CSF

All the SiMoA (Single Molecular Array) assays were performed on the Quanterix SR-X analyzer (Quanterix) as previously described [65]. The concentrations of Tau, Aβ 40 and Aβ 42 in CSF were measured using the Simoa® Neurology 3-Plex A Advantage Kit (catalog #101995) according to the manufacturer’s instructions. The concentrations of pTau-181 in CSF were measured using the Simoa® pTau-181 Advantage V2 Kit (catalog #103714) according to the manufacturer’s instructions. CSF samples were diluted 1:100–400. All measurements were carried out in one round of experiment using the same batch of reagents.

### Western blot analysis

Total protein from mouse brain tissue samples were extracted with lysis buffer (50 mM Tris, 40 mM NaCl, 1 mM EDTA, 0.5% Triton X-100, 1.5 mM Na_3_VO_4_, 50 mM NaF, 10 mM sodium pyrophosphate and 10 mM sodium β-glycerophosphate, pH 7.0, supplemented with a cocktail of protease inhibitors). Protein concentration of samples was measured using a bicinchoninic acid protein assay kit (Thermo Fisher Scientific). Equal protein amounts (30 to 50 μg) were loaded for SDS-PAGE and transferred to a polyvinylidene difluoride or nitrocellulose membrane. After blocking the nonspecific site with 5% nonfat milk for 1 h, the membrane was incubated with a specific primary antibody overnight at 4 °C and then incubated with horseradish peroxidase–conjugated secondary antibody for 1 h at room temperature. The immune blotting signals were visualized with an enhanced chemiluminescence (ECL) kit (Thermo Fisher Scientific, catalog no. 32209) using a chemiluminescent imaging system (Bio red). Digital images were quantified using densitometric measurement with ImageJ software.

### Real-time PCR

Total RNA from brain tissue was extracted using TRIzol Reagent (Invitrogen, Carlsbad, CA, USA) according to the manufacturer’s instructions. The concentration of RNA was quantified by the NanoDrop 2000c Spectrophotometer (Thermo Fisher Scientific, Rockford, IL). The first-strand cDNA was synthesized from 1 μg of total RNA using Hifair One Step RT-qPCR Kit (YEASEN Biotech, Shanghai, China). The quantitative PCR experiments were conducted on a QuantStudio Real-Time PCR system (Applied Biosystems) using Hieff® qPCR SYBR Green Master Mix (YEASEN Biotech) with gene-specific primers. Glyceraldehyde-3- phosphate dehydrogenase (GAPDH) was used as an internal control for normalization. All reactions were performed in triplicate with three independent experiments, and the relative expression of mRNA levels was calculated using the 2^−△△Ct^ method.

### Immunofluorescence staining, Thioflavin S and cold PET tracer staining

Mice were deeply anesthetized and perfused with ice-cold PBS followed by 4% PFA. The whole brain was dissected, fixed in 4% PFA overnight, dehydrated in 30% sucrose at 4 °C, embedded in OCT (optimal cutting temperature compound) (Sakura Finetek), and processed for cryosections at 20 μm. The cryosections were permeabilized and blocked in blocking buffer [0.4% Triton X-100 and 5% normal bovine serum albumin (BSA) in PBS] for 1 h at room temperature and overnight with primary antibodies overnight at 4 °C. After washing with PBS, sections were incubated with secondary antibodies labeled with Alexa Fluor 488 or 594 (Jackson ImmunoResearch Laboratories; 1:1000) for 1 h at room temperature, stained with 4′,6-diamidino-2-phenylindole (DAPI) for 10 min, washed three times in PBS, and then mounted in Mounting Medium. For Thioflavin S (ThS), AV45 with Aβ or AV1451 with AT8 double staining, slides were rinsed in PBS after finishing the Aβ or AT8 first antibody and the second antibody staining. Freshly dissolved ThS, AV45 or AV1451 in 50% Ethanol and incubated the slides in ThS, AV45 or AV1451 solution at room temperature for 7 min in the dark. Then wash the slides in PBS for three times. Last, coverslips were mounted on glass slides and imaged using a confocal microscope (LSM 980, Zeiss).

### Immunohistochemistry

Immunohistochemistry (IHC) was carried out following the peroxidase protocol. Briefly, tissue sections were deparaffinized in xylene, hydrated through descending ethanol concentrations, and endogenous peroxidase activity was eliminated by incubation in 3% hydrogen peroxide in methanol for 5 min. After antigen-retrieval in boiling sodium citrate buffer (10 mM), the sections were incubated with primary antibodies for overnight at 4 °C. The signal was developed using Mouse and Rabbit Specific HRP/DAB detection IHC kit (Abcam). Images were acquired using Olympus VS120 virtual microscopy slide scanning system.

### ELISA assay of Aβ, Tau and cytokines

Mouse brain samples were homogenized in lysis buffer and centrifuged at 16,000 g for 20 min at 4 °C. The supernatant was for Aβ, Tau, IL6 and TNFα analyzed by ELISA kits according to the manufacturer’s instructions. The insoluble protein including Aβ and Tau aggregates were extracted following the protocol as described in previous work [66, 67] and then were analyzed by ELISA kits according to the manufacturer’s instructions.

### Isolation of Sarkosyl insoluble Tau aggregates in brain tissue

Sarkosyl insoluble Tau were extracted as described previously [67]. Briefly, mice were euthanized by cervical dislocation in order to preserve the metabolic environment of the brain and to prevent artifacts that could alter the biochemical profiles of tau protein. Mouse brains were bisected down the midline to yield two hemispheres. The forebrain including cerebral cortex and hippocampus was separated from the hemisphere by a razor blade, and homogenized in ten volumes of Tris-buffered saline (TBS) buffer (50 mM Tris/HCl, pH 7.4, 274 mM NaCl, 5 mM KCl) containing the protease and phosphatase inhibitors with a mechanical homogenizer. Centrifuge at 27,000 g for 20 min at 4 °C. Keep the supernatant as soluble fraction of Tau. From the pellet, homogenized with 5 vol. (v/w) of high salt / sucrose buffer (0.8 M NaCl, 10% sucrose, 10 mM Tris-HCl, pH 7.4, 1 mM EGTA, 1 mM PMSF). Centrifuge at 27,000 g for 20 min at 4 °C. The supernatant was adjusted to 1% Sarkosyl and incubated for 1 h at 37 °C on orbital shaker, and centrifuged at 150,000 g for 1 h at 4 °C. The pellet was resuspended with TE buffer (10 mM Tris-HCl, pH 8.0, 1 mM EDTA.) as Sarkosyl insoluble fraction of Tau aggregates.

### Isolation of insoluble Aβ aggregates in brain tissue

Aβ species were extracted from brain homogenates as previously described [66]. In brief, equal volumes of 0.4% diethylamine (DEA) to brain homogenate were subjected to ultraspeed centrifugation at 135,000 g for 1 h at 4 °C. After neutralization, supernatant was collected and used as soluble Aβ species. The remaining pellet was then dissolved in formic acid (FA), subjected again to ultraspeed centrifugation at 109,000 g for 1 h at 4 °C, and, after neutralization, the supernatant was collected and used to analyze insoluble Aβ species.

### Transduction of Tau aggregates

HEK293-K18 cells that stably expressed tau repeat domain were seeded in 6-well plates. Twenty-four h later, Tau aggregates extracted from 3xTg and Thy1-ApoE4/C/EBPβ mice (2 μg) were transfected into HEK293-K18 cells using lipofectamine-3000 (Invitrogen) according to the manufacturer’s instructions. Eighteen h later, cells were fixed with 4% PFA in PBS for 10 min and then stained with DAPI for 5 min. Coverslips were mounted, sealed with nail polish, and placed at 4 °C prior to analysis under Zeiss confocal imaging system (LSM 900). To quantify percent cells positive for Tau inclusions, a total of 10 fields were analyzed. The percentage of cells with inclusions were calculated based on the number of DAPI positive nuclei.

### Protease K digestion

Proteinase K (Roche) was diluted in PBS to a final concentration of 1 mg/mL and single-use aliquots were stored at −80 °C. Tau aggregates (5 µg) was added to proteinase K at a concentration of 1 µg/mL (diluted in PBS) for a final volume of 20 µL. After different time of digestion, the mixture was subjected to SDS-PAGE gel for Tau blotting.

### Imaging by transmission electron microscopy

An aliquot of 5 μL of Tau or Aβ aggregates isolated from 3xTg and Thy1-ApoE4/C/EBPβ mice were deposited on carbon-coated copper grids (Electron Microscopy Sciences, Washington, PA,USA), and then negatively stained with 2% Phosphotungstic acid for 1 min. After 3 times of washing and air-dried for 5 min, grids were examined using a Hitachi-7100 transmission electron microscope operated at 80 kV.

### Primary cortical neuronal cultures

For primary cortical neuron culture, the cortex of embryonic day 18 Sprague–Dawley rat embryos was dissected and digested with 0.05% trypsin for 10 min at 37 °C. The digestion was stopped by adding 20% fetal bovine serum and then centrifuged at 1000 × g for 5 min. Neurons were seeded in 6-well dishes with coverslips pre-treated with 100 µg/mL poly-D-lysine (Sigma Aldrich) after addition of neuronal plating medium containing DMEM/F12 with 10% fetal bovine serum. After 6 h of incubation, medium was replaced by Neurobasal medium (Gibco) containing serum-free B-27 (Invitrogen) and GlutaMAX (Invitrogen) and cultured in a humidified incubator with 5% CO2 at 37 °C. At day of 15 in vitro culture, neurons were treated with Aβ or Tau aggregates extracted from 3xTg and Thy1-ApoE4/C/EBPβ mice.

### Animals and hippocampal injections

APP/PS1 (2 months old) and Tau P301S (3 months old) mice were used for hippocampal injection of Aβ and Tau aggregates from 3xTg and Thy1-ApoE4/C/EBPβ mice, respectively. Mice were anesthetized with isoflurane and were unilaterally injected into the hippocampus (−2.5 mm posterior, ±2 mm lateral, −1.8 mm ventral from bregma). Either 2 μl of 5 mg/mL Aβ aggregates or 2 μl of 2.5 mg/mL Tau aggregates were delivered by using a micro syringe pump (WPI, UMP3/Micro2T, USA) with a 10 μL Hamilton syringe. The syringe was kept still for 10 min till the end of injection to allow diffusion. After 2-month of injection, mice were sacrificed for histological analysis. All protocols involving experimental animal were approved by the institutional animal care and use committee at the SIAT.

### AEP activity assay

Total protein from brain tissue homogenates (10 μg) were incubated with 200 μl reaction buffer (0.1% CHAPS, 20 mM citric acid, 60 mM Na2HPO4, 1 mM EDTA and 1 mM DTT, pH 6.0) containing 20 μM AEP substrate Z-Ala-Ala-Asn-AMC (4-methyl-7-coumarylamide) (Bachem). The fluorescence released by substrate AMC cleavage was quantified by measuring at 460 nm in a fluorescence plate multi-mode reader (Biotex, Synergy HTX) at 37 °C for 2 h in kinetic mode.

### Thioflavin T fluorescence assay

To assess coaggregation of mouse Aβ with human ApoE4 / ApoE3, monomeric mouse Aβ42 (10 μM) was incubated with purified ApoE4 / ApoE3 (0.1 μM) in ThT fluorescence assay buffer (50 mM sodium phosphate buffer, pH 7.4, 50 mM NaCl, 10 μM ThT and 0.01% sodium azide) at 37 °C with an orbital shaking. Real-time ThT fluorescence measurements were recorded every 10 min for 5 h at excitation and emission wavelengths of 440 nm and 490 nm on a Biot Tek microplate reader. As a positive control, ThT fluorescence measurements were also performed using human Aβ42 aggregation as described above.

### MTT assay

SH-SY5Y cells were seeded into 96-well culture plates at a density of 100,000 cells/well in growth medium supplemented with 10% serum. The cells were treated with aggregation of mouse Aβ42, mouse Aβ42/ApoE4, and mouse Aβ42/ApoE3 for 24 h and then used for cell viability assay using the Cell Counting Kit-8 (Yeasen, China) following the manufacturer’s protocol. The absorption was read at 570 nm using micro-plate spectrophotometer (Bio Tek).

### Statistical analysis

Statistical analyses were performed with the SPSS version 13.0 software 261 package (SPSS, Chicago, IL, USA) for Windows. All data are presented as means ± SD unless otherwise stated. When only two groups were compared, the statistical differences were assessed with the double-sided Student’s t test. Sample size was determined by Power and Precision (Biostat).The number of samples per group (*n* ≥ 6) is stated in the figure legends. Comparisons among multiple groups were performed using one-way analysis of variance (ANOVA) with Tukey’s post hoc test. Two-way ANOVA was used for analysis of multiple groups with Tukey’s multiple comparison post hoc test. For all experiments, *p ≤ 0.05 was considered a significant difference.

## Supplementary information

Supplementary file

## Data Availability

All data generated or analysed during this study are included in this published article and its supplementary information files. The datasets of the current study are available from the corresponding author on reasonable request.
