# Supplementary material for: Thy1-ApoE4/C/EBPβ double transgenic mice act as a sporadic model with Alzheimer’s disease
Source: Mol Psychiatry. 2024 Apr 24;29(10):3040–55. doi: 10.1038/s41380-024-02565-x (PMC11449781; doi:10.1038/s41380-024-02565-x)
Supplement: Supplementary file 1 — Supplementary file [file 41380_2024_2565_MOESM1_ESM.pdf]

Supplementary Information for

Thy1-ApoE4/C/EBPβ Double Transgenic Mice Act as a Sporadic Model with Alzheimer's Disease

By

Zhengjiang Qian[1], ZhiHao Wang[2], Bowei Li[3], Xin Meng[1], Zhonghua Kuang[4], Yanjiao Li[1], Yongfeng Yang[4] and Keqiang Ye[1,*]

This PDF file includes:

Supplementary Fig. 1 to Fig. 8

Supplementary Fig. 1

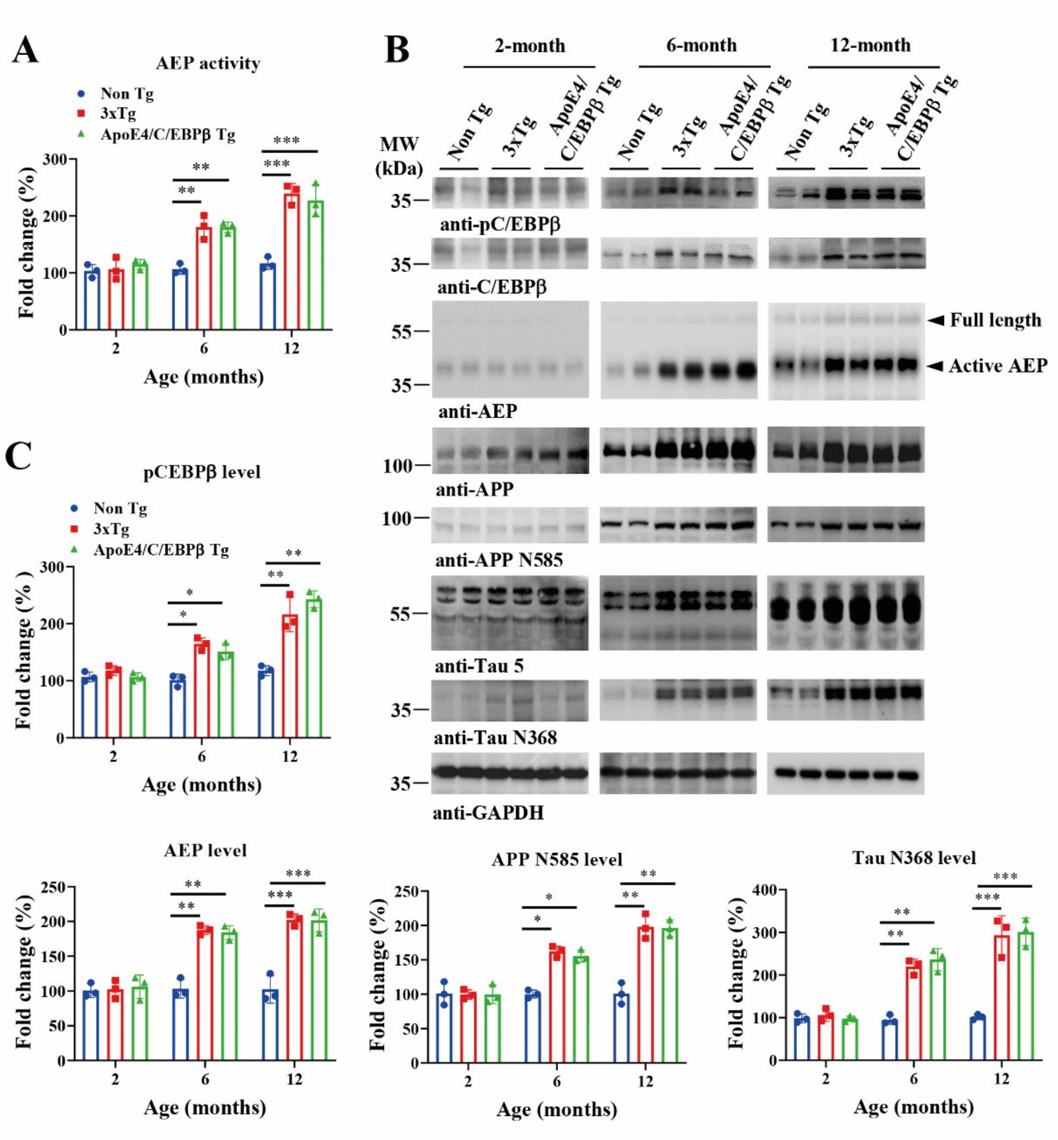

Supplementary Fig. 1 C/EBPβ/AEP signaling activation in 3xTg and Thy1-ApoE4 /C/EBPβ Tg mice

A. AEP enzymatic activity in the brains of 3xTg and Thy1-ApoE4/C/EBPβ Tg mice at 2-, 6- and 12-month of age. (n = 3, $^{**}p < 0.01$, $^{***}p < 0.001$, compared with Non Tg).

B. Western blotting assays of C/EBPβ, AEP and its downstream targets in the brains of 3xTg and Thy1-ApoE4/C/EBPβ Tg mice at 2-, 6- and 12-month of age.

C. Relative quantification of protein expression in (B). (n = 3, $^{**}p < 0.01$, $^{***}p < 0.001$, compared with Non Tg).

Supplementary Fig. 2

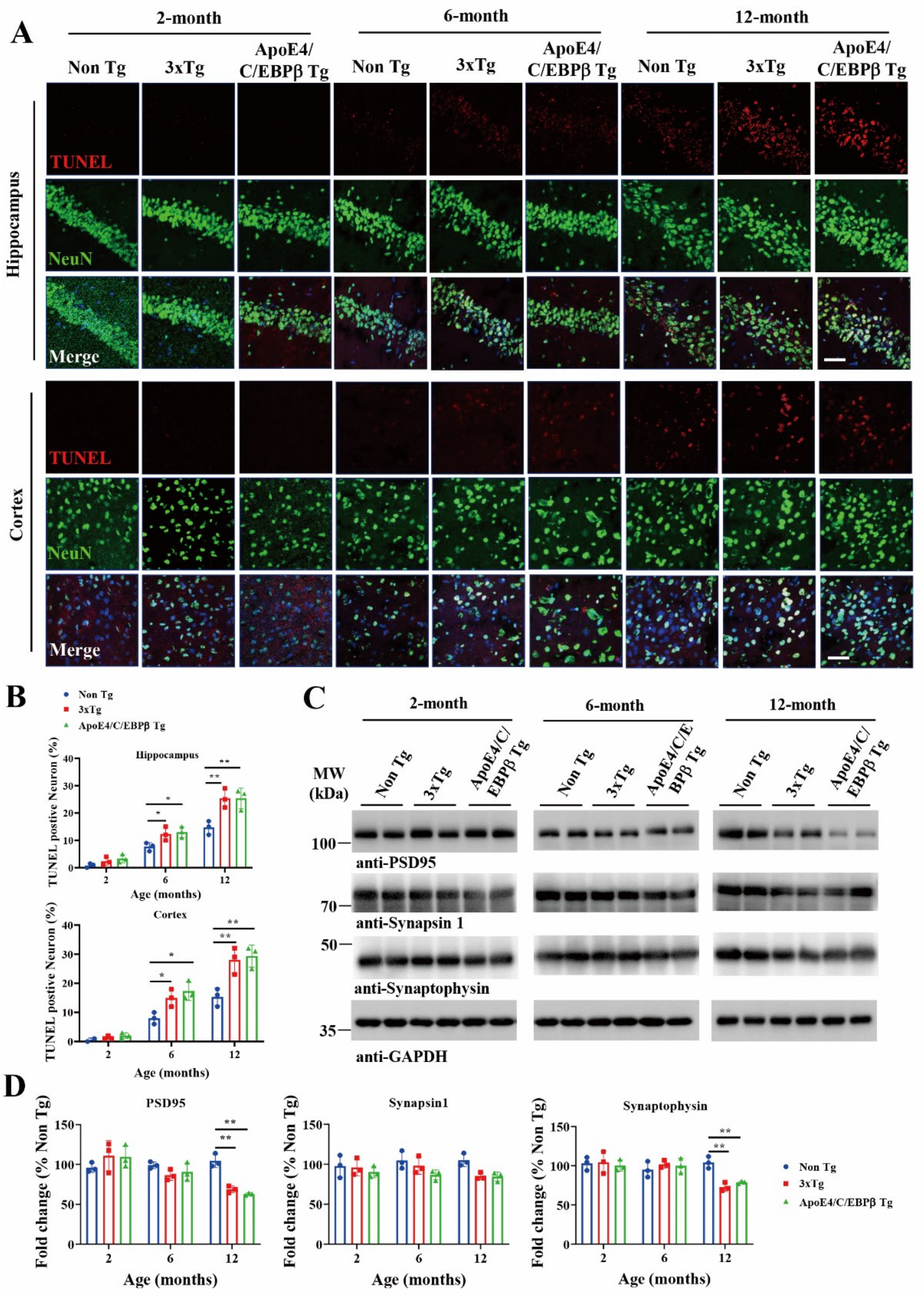

Supplementary Fig. 2 Age-dependent neuronal loss in 3xTg and Thy1-ApoE4/C/EBPβ Tg mice.

A. Immunostaining of NeuN and TUNEL in hippocampus (top panels) and cortex (bottom panels) of 3xTg and Thy1-ApoE4/C/EBPβ Tg mice at 2-, 6- and 12-month of age. Scale bar, 20 μm.

B. Relative quantification of TUNEL positive NeuN in (A). (n = 6, *$p < 0.05$, **$p < 0.01$, compared with Non Tg).

C. Representative immunoblot images showing the expression of synaptic markers in 3xTg and Thy1-ApoE4/C/EBPβ Tg mice at 2-, 6- and 12-month of age.

D. Relative quantification of protein levels in (C). (n = 3, ** $p < 0.01$, compared with Non Tg).

Supplementary Fig. 3

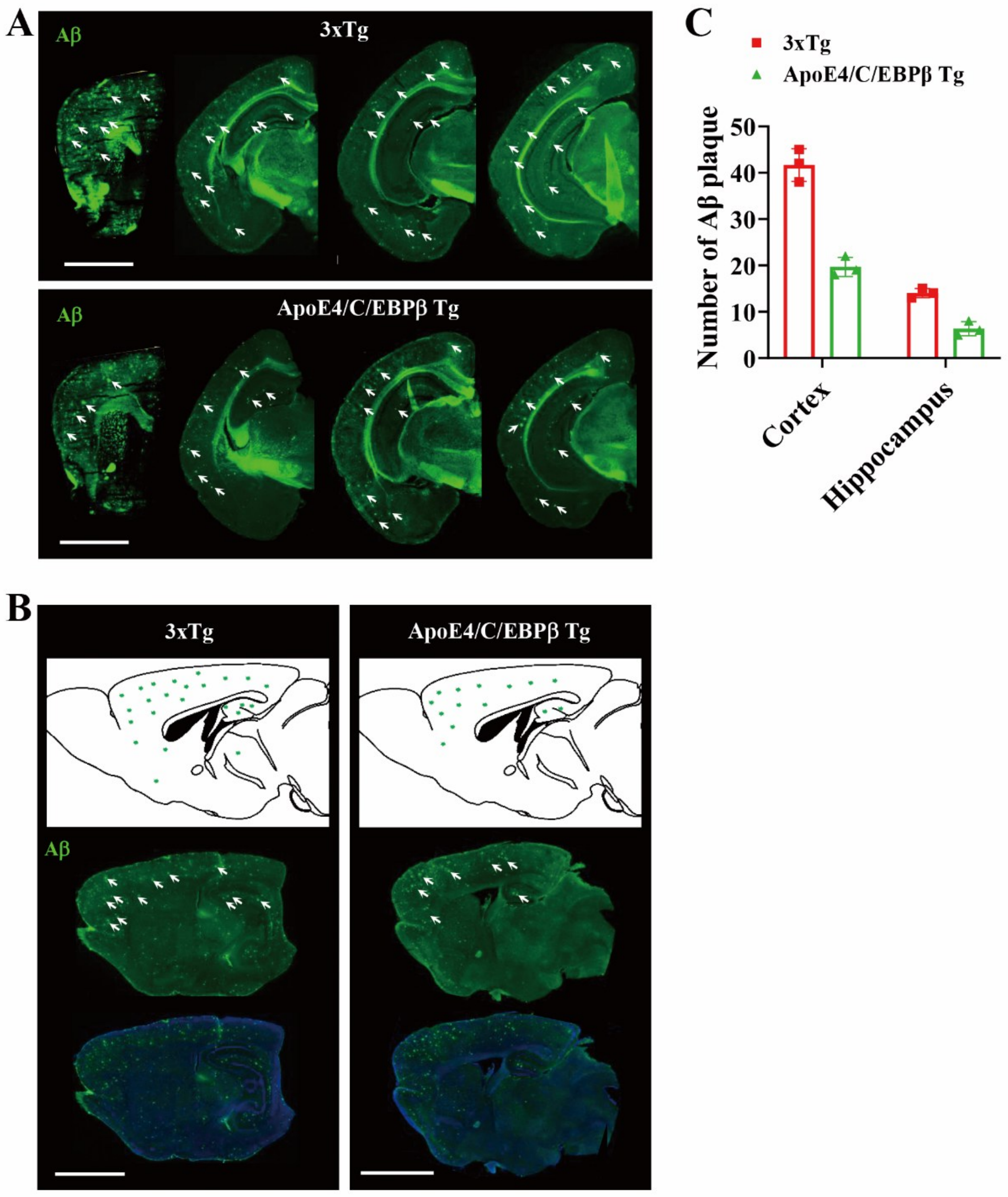

Supplementary Fig. 3 Representative image of distribution of Aβ pathology in 3xTg and Thy1-ApoE4/C/EBPβ Tg mice.

A. Representative coronal section of Aβ plaque in 3xTg and Thy1/ApoE3/C/EBPβ transgenic mice at the age of 12-month. Scale bar 500 µm

B. Representative sagittal section of Aβ plaque in 3xTg and Thy1/ApoE3/C/EBPβ transgenic mice at the age of 12-month. Scale bar 500 µm.

C. Relative quantification of Aβ plaque numbers in cortex and hippocampus of 3xTg and Thy1/ApoE3/C/EBPβ transgenic mice at the age of 12-month.

Supplementary Fig. 4

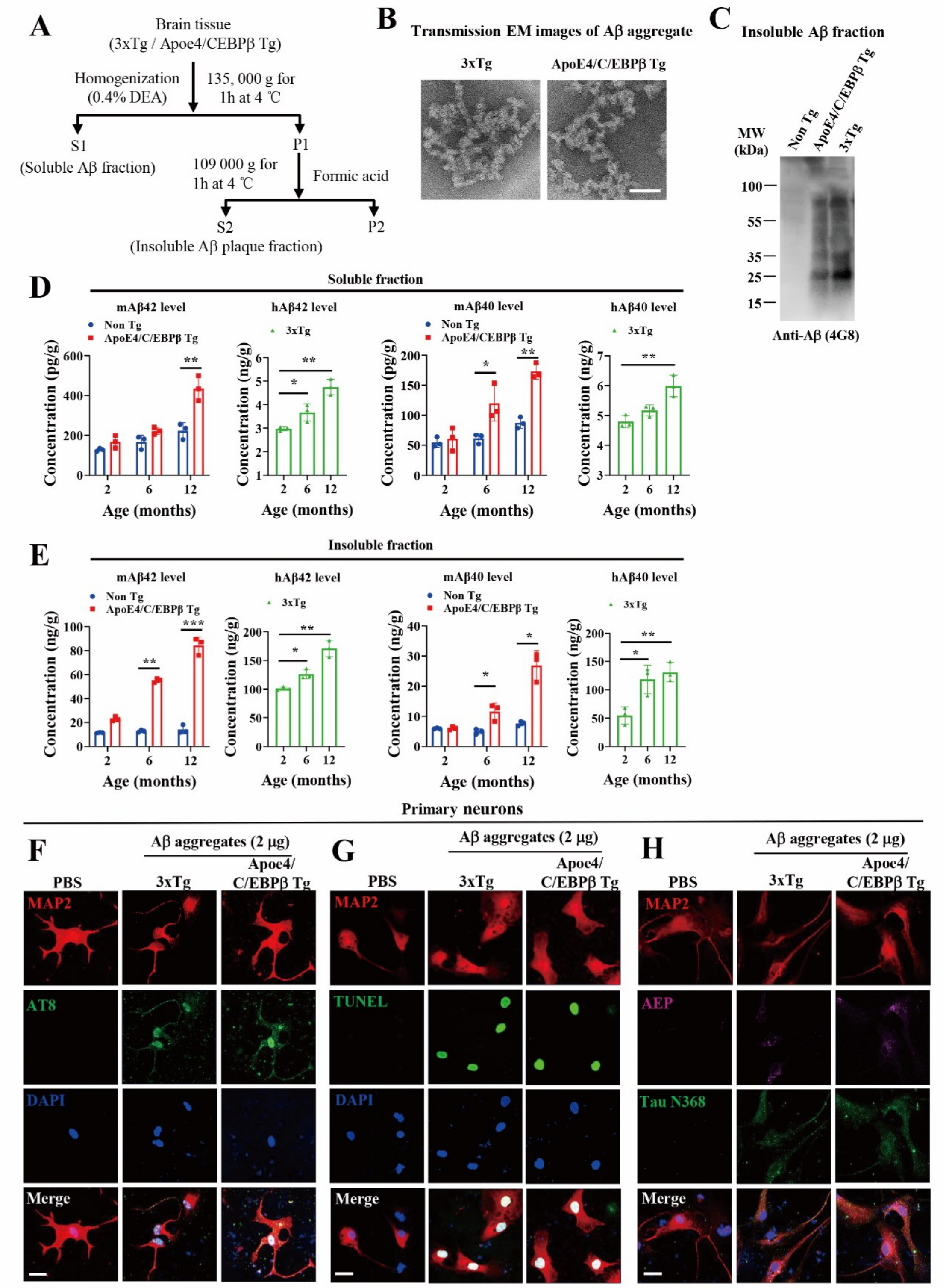

Supplementary Fig. 4 Characterization of Aβ aggregates in 3xTg and Thy1-ApoE4/C/EBPβ Tg mice.

A. Diagram showing the isolation of soluble and insoluble Aβ in the brain tissues of 3xTg and Thy1-ApoE4/C/EBPβ Tg mice.

B. Representative electron microscope images of insoluble Aβ fraction extracted from the brains of 3xTg and Thy1-ApoE4/C/EBPβ Tg mice. Scale bar 100 nm.

C. Western blotting assay of insoluble Aβ fraction extracted from the brains of 3xTg and Thy1-ApoE4/C/EBPβ Tg mice.

D. ELISA assay of soluble Aβ40 and Aβ42 levels in the brains of 3xTg and Thy1-ApoE4/C/EBPβ Tg mice at different ages. (n = 3, *$p < 0.05$, **$p < 0.01$).

E. ELISA assay of insoluble Aβ40 and Aβ42 levels in the brains of 3xTg and Thy1-ApoE4/C/EBPβ Tg mice at different ages. (n = 3, *$p < 0.05$, **$p < 0.01$, ***$p < 0.001$).

F-H. Representative immunostaining images of AT8 (F), TUNEL (G), AEP and Tau N368 (H) in primary rat neurons treated with Aβ aggregates (2 μg) extracted from the brains of 3xTg and Thy1-ApoE4/C/EBPβ Tg mice. Scale bar 20 μm.

Supplementary Fig. 5

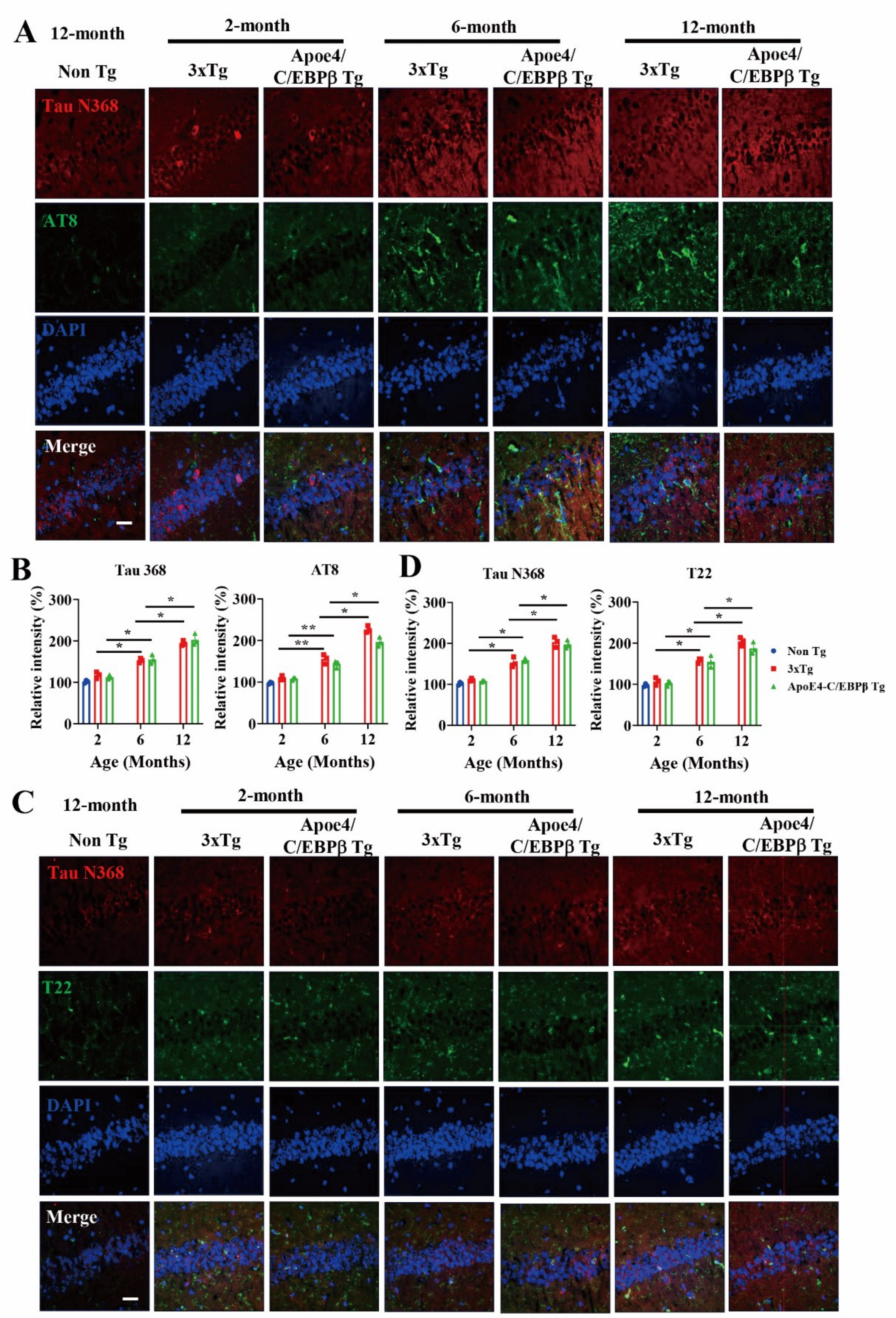

Supplementary Fig. 5 Age-dependent increases of Tau pathology in 3xTg and Thy1-ApoE4/C/EBPβ Tg mice.

A. Co-staining of Tau N368 and AT8 in the hippocampus of 3xTg and Thy1-ApoE4/C/EBPβ Tg mice at 2-, 6- and 12-month of age. Scale bar 20 μm.

B. Quantification of AT8 and Tau N368 fluorescent signals in (A). (n = 3, *$p < 0.05$, **$p < 0.01$).

C. Co-staining of Tau N368 and AT22 in the hippocampus of 3xTg and Thy1-ApoE4/C/EBPβ Tg mice at 2-, 6- and 12-month of age. Scale bar 20 μm

D. Quantification of T22 and Tau N368 fluorescent signals in (C). (n = 3, *$p < 0.05$).

Supplementary Fig. 6

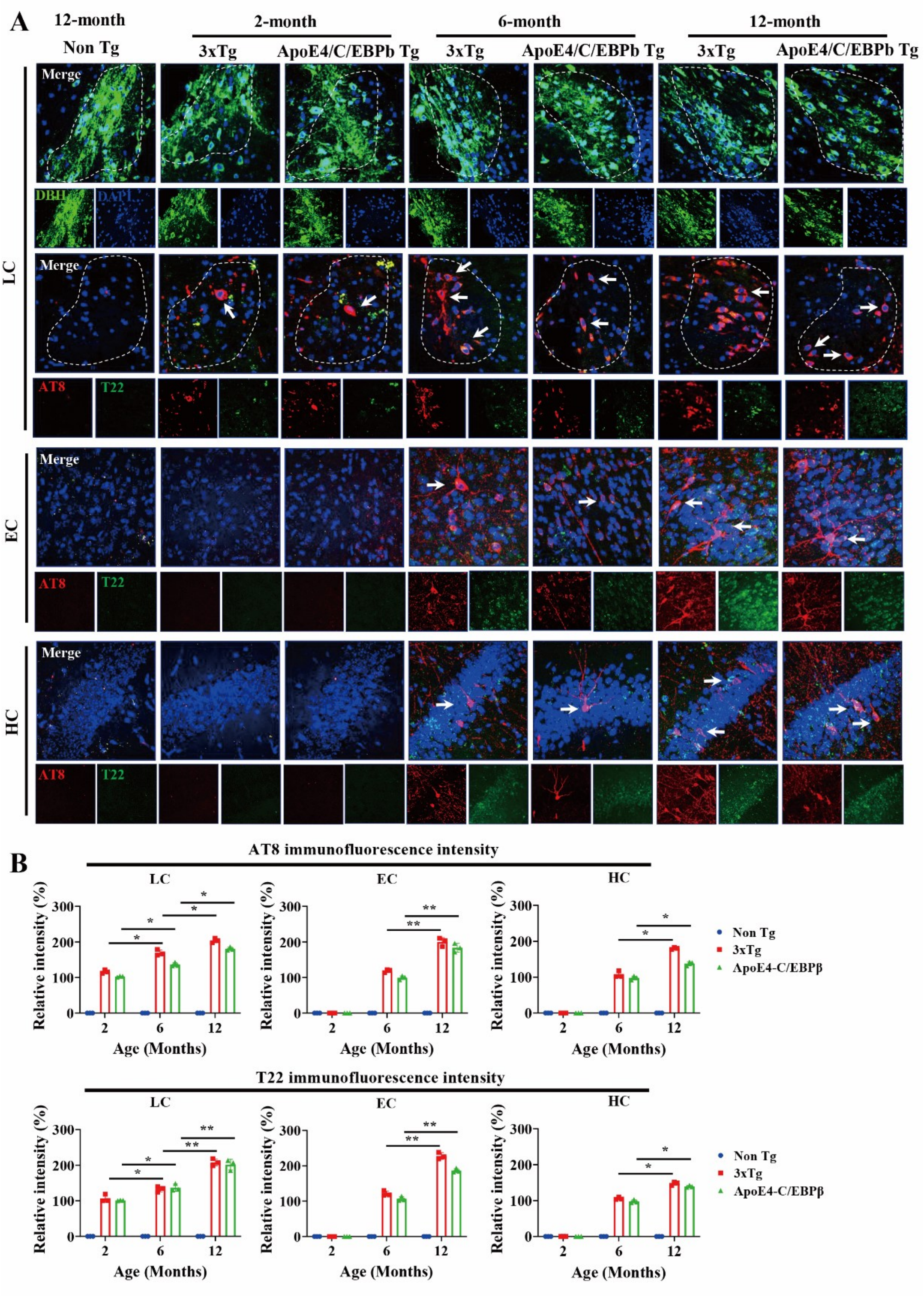

Supplementary Fig. 6 Tau spreading in 3xTg and Thy1-ApoE4/C/EBPβ Tg mice

A. Representative AT8 and T22 immunostaining images in LC, EC and HC of 3xTg and Thy1-ApoE4/C/EBPβ Tg mice at 2-, 6- and 12-month of age. LC, Locus coeruleus; EC, entorhinal cortex; HC, hippocampus.

B. Quantification of AT8 and T22 fluorescent signals in LC, EC and HC. ($n = 3$, *$p < 0.05$, **$p < 0.01$)

Supplementary Fig. 7

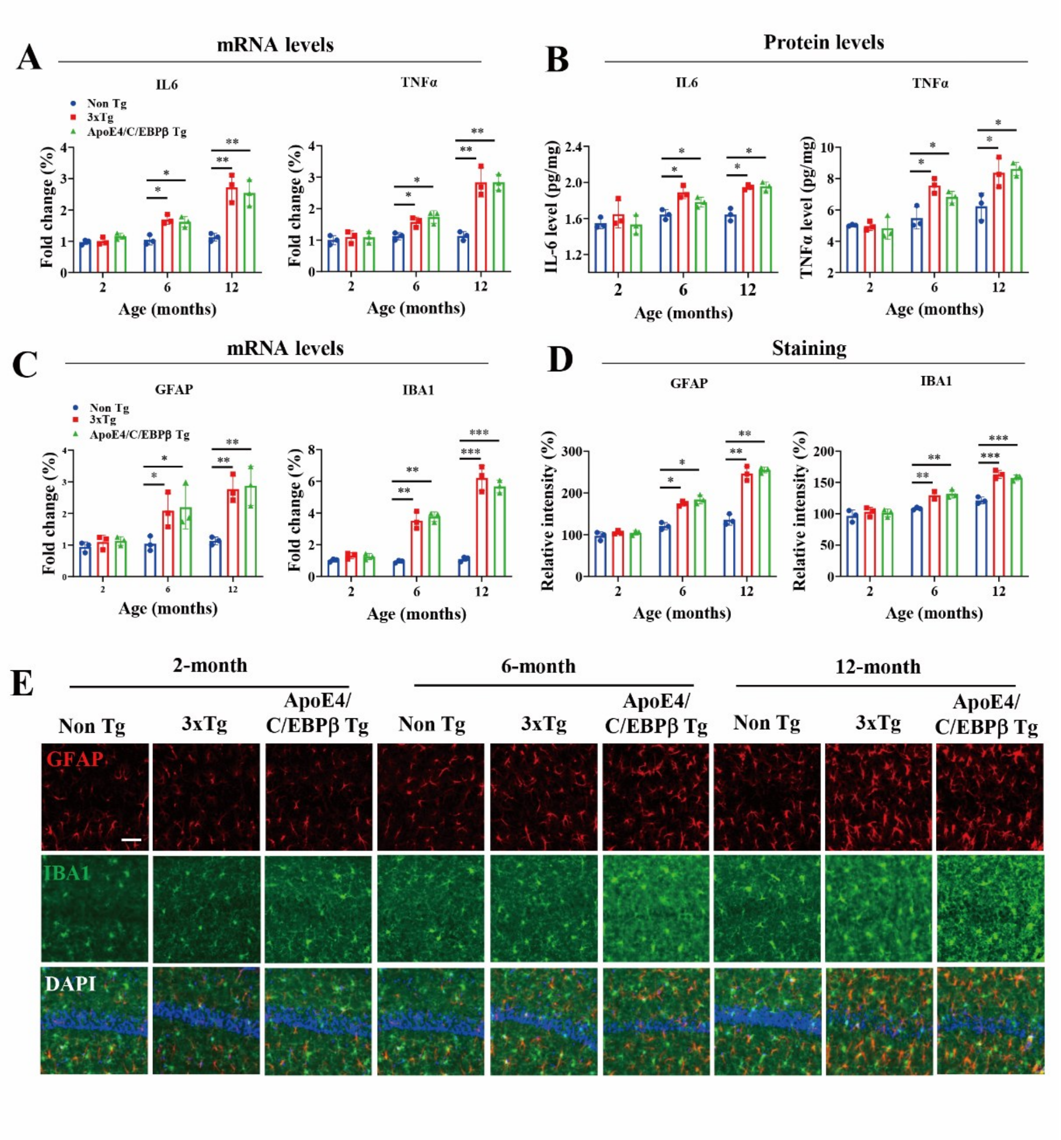

Supplementary Fig. 7 Neuroinflammation in 3xTg and Thy1-ApoE4/C/EBPβ Tg mice

A. The mRNA expression of IL-6 (left panel) and TNF-α (right panel) in the brain of 3xTg and Thy1-ApoE4/C/EBPβ Tg mice at 2-, 6- and 12-month of age. (n = 3, *$p < 0.05$, **$p < 0.01$, compared with Non Tg).

B. ELISA analysis of mouse IL-6 (left panel) and TNF-α (right panel) in the brain tissues of 3xTg and Thy1-ApoE4/C/EBPβ Tg mice at 2-, 6- and 12-month of age. (n = 3, *$p < 0.05$, compared with Non Tg).

C. The mRNA expression of GFAP (left panel) and Iba1 (right panel) in the brains of 3xTg and Thy1-ApoE4/C/EBPβ Tg mice at 2-, 6- and 12-month of age. (n = 3, *$p < 0.05$, **$p < 0.01$, ***$p < 0.01$, compared with Non Tg).

D. Relative quantification of GFAP (left panel) and Iba1 (right panel) fluorescence intensity in (E). (n = 3, *$p < 0.05$, compared with vehicle).

E. Immunostaining of GFAP and Iba1 in hippocampus of 3xTg and Thy1-ApoE4/C/EBPβ Tg mice at 2-, 6- and 12-month age. Scale bar, 100 μm.

Supplementary Fig. 8

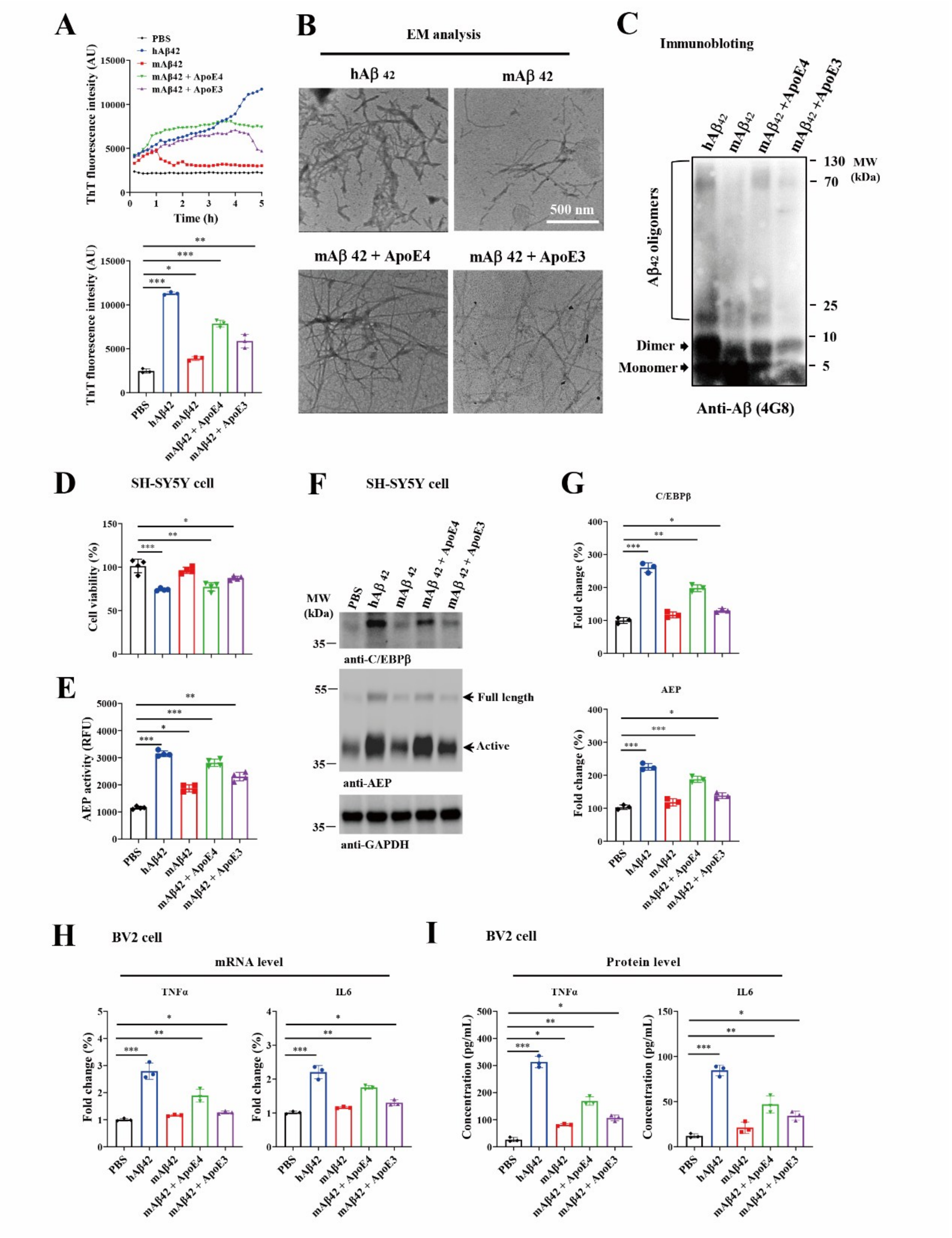

Supplementary Fig. 8 ApoE4 promotes mouse Aβ aggregation and neurotoxicity

A. Representative curves of the Thioflavin T (ThT) fluorescence assays following ApoE4 and mouse Aβ42 (mAβ42) co-incubation. Human Aβ42 (hAβ42), mAβ42 alone and ApoE3 / mAβ42 co-incubation were taken as controls. The ThT assay was independently repeated three times.

B. TEM analysis of ApoE4 and mAβ42 incubation at 37 °C for 96 h. Similar results were observed in four independent experiments. Scale bar, 500 nm.

C. Immunoblotting analysis showing ApoE4-mediated enhancement of mAβ42 oligomer formation *in vitro*.

D and E. Effects of ApoE4 / mAβ42 incubation on cell viability (D) and AEP activity in SH-SY5Y cells. (n = 3, *$p < 0.05$, **$p < 0.01$, ***$p < 0.01$, compared with PBS).

F. Immunoblotting analysis showing ApoE4 / mAβ42 incubation activate C/EBPβ and AEP expression in SH-SY5Y cells.

G. Relative quantification of protein expression in (F). (n = 3, *$p < 0.05$, **$p < 0.01$, ***$p < 0.001$, compared with PBS).

H and I. Effects of ApoE4 / mAβ42 incubation on TNFα and IL-6 at mRNA (H) and protein (I) levels in microglial BV2 cells. (n = 3, *$p < 0.05$, **$p < 0.01$, ***$p < 0.001$, compared with PBS).
